# YY-1224, a terpene trilactone-strengthened *Ginkgo biloba*, attenuates neurodegenerative changes induced by β-amyloid (1-42) or double transgenic overexpression of APP and PS1 via inhibition of cyclooxygenase-2

**DOI:** 10.1186/s12974-017-0866-x

**Published:** 2017-04-27

**Authors:** Zheng-Yi Li, Yoon Hee Chung, Eun-Joo Shin, Duy-Khanh Dang, Ji Hoon Jeong, Sung Kwon Ko, Seung-Yeol Nah, Tae Gon Baik, Jin Hyeong Jhoo, Wei-Yi Ong, Toshitaka Nabeshima, Hyoung-Chun Kim

**Affiliations:** 1 Neuropsychopharmacology and Toxicology Program, College of Pharmacy, Kangwon National University, Chunchon, 24341 Republic of Korea; 2 Department of Anatomy, College of Medicine, Chung-Ang University, Seoul, 06974 Republic of Korea; 3 Department of Pharmacology, College of Medicine, Chung-Ang University, Seoul, 06974 Republic of Korea; 4 Department of Oriental Medical Food and Nutrition, Semyung University, Jecheon, 27136 Republic of Korea; 5 Ginsentology Research Laboratory and Department of Physiology, College of Veterinary Medicine and Bio/Molecular Informatics Center, Konkuk University, Seoul, 05029 Republic of Korea; 6 R&D Center, Yuyu Pharma, Seoul, 04598 Republic of Korea; 7 Department of Psychiatry, Medical School, Kangwon National University, Chunchon, 24341 Republic of Korea; 8 Department of Anatomy, National University of Singapore, Singapore, 119260 Singapore; 9 Nabeshima Laboratory, Graduate School of Pharmaceutical Sciences, Meijo University, Nagoya, 468-8503 Japan

**Keywords:** Terpene trilactone-strengthened *G. biloba*, Platelet-activating factor, Peroxisome proliferators-activated receptor γ, Microglia, APPswe/PS1dE9 transgenic mice, Cyclooxygenase-2 knockout mice

## Abstract

**Background:**

*Ginkgo biloba* has been reported to possess free radical-scavenging antioxidant activity and anti-inflammatory properties. In our pilot study, YY-1224, a terpene trilactone-strengthened extract of *G. biloba*, showed anti-inflammatory, neurotrophic, and antioxidant effects.

**Results:**

We investigated the pharmacological potential of YY-1224 in β-amyloid (Aβ) (1-42)-induced memory impairment using cyclooxygenase-2 (COX-2) knockout (−/−) and APPswe/PS1dE9 transgenic (APP/PS1 Tg) mice. Repeated treatment with YY-1224 significantly attenuated Aβ (1-42)-induced memory impairment in COX-2 (+/+) mice, but not in COX-2 (−/−) mice. YY-1224 significantly attenuated Aβ (1-42)-induced upregulation of platelet-activating factor (PAF) receptor gene expression, reactive oxygen species, and pro-inflammatory factors. In addition, YY-1224 significantly inhibited Aβ (1-42)-induced downregulation of PAF-acetylhydrolase-1 (PAF-AH-1) and peroxisome proliferator-activated receptor γ (PPARγ) gene expression. These changes were more pronounced in COX-2 (+/+) mice than in COX-2 (−/−) mice. YY-1224 significantly attenuated learning impairment, Aβ deposition, and pro-inflammatory microglial activation in APP/PS1 Tg mice, whereas it significantly enhanced PAF-AH and PPARγ expression. A preferential COX-2 inhibitor, meloxicam, did not affect the pharmacological activity by YY-1224, suggesting that the COX-2 gene is a critical mediator of the neuroprotective effects of YY-1224. The protective activity of YY-1224 appeared to be more efficacious than a standard *G. biloba* extract (Gb) against Aβ insult.

**Conclusions:**

Our results suggest that the protective effects of YY-1224 against Aβ toxicity may be associated with its PAF antagonistic- and PPARγ agonistic-potential as well as inhibition of the Aβ-mediated pro-inflammatory switch of microglia phenotypes through suppression of COX-2 expression.

**Electronic supplementary material:**

The online version of this article (doi:10.1186/s12974-017-0866-x) contains supplementary material, which is available to authorized users.

## Background

Alzheimer’s disease (AD) is the most common neurodegenerative disorder in the elderly and is associated with progressive memory loss and cognitive dysfunction [1–4]. Although the precise cause of the AD is unknown, β-amyloid peptide (Aβ)-induced neurotoxicity, tau pathology, and neuroinflammatory responses by microglia are thought to contribute to the pathogenesis of AD [1, 5]. Several studies have suggested that treatment with nonsteroidal anti-inflammatory drugs (NSAIDs) attenuates the loss of cognitive function and decreases the risk of developing AD [6, 7]. NSAIDs act by inhibiting cyclooxygenase (COX), which is the rate-limiting enzyme for the conversion of arachidonic acid into inflammatory mediators, which include prostaglandin E2 (PGE2). COX-2 enzymatically mediates the inflammatory response, and its expression is significantly increased in the AD brain [8, 9].

Platelet-activating factor (PAF) is a highly potent inflammatory mediator and a potential neurotoxin [10]. It plays an important role in excitotoxicity, production of free radicals and nitric oxide (NO), and regulation of pro-inflammatory cytokine genes [11–14]. The biological action of PAF is mostly mediated by binding to its G protein-coupled membrane-associated receptor (PAFR) [15]. In vitro studies have shown that activation of epidermal PAFR results in biosynthesis of COX-2 [16]. Both PAFR and COX-2 are involved in memory processing in vivo [17, 18]. PAF is a short-lived molecule due to its rapid degradation by PAF acetylhydrolase (PAF-AH; EC 3.1.1.47) [19]. PAF-AH is an enzyme that hydrolyzes an acetyl ester at the sn-2 position of PAF, converting it into its inactive metabolite, 1-*O*-alkyl-sn-glycero-3-phosphocholine (lysoPAF) [20]. Three isoforms of PAF-AH were identified: plasma PAF-AH and PAF-AH II and I [21].

NSAIDs may regulate gene expression via their interaction with peroxisome proliferators-activated receptors (PPARs). There are three PPAR isoforms: PPAR α, β, and γ. PPARγ, in particular, has been implicated in inflammation and neurodegeneration [22]. The messenger RNA (mRNA) levels of PPARγ are increased in AD patients [23], suggesting that PPARγ may play an important role in modulating the pathophysiology of AD. The PPARγ isoform can be activated by NSAIDs, and its activation in the microglia suppressed the expressions of inflammatory cytokines, inducible NO synthase (iNOS), and COX-2 [24]. Aβ-induced activation of microglia was suppressed by a PPARγ agonist in vitro [24]. Furthermore, PPARγ agonists significantly decreased Aβ42 levels in vivo [25].

It has been demonstrated that a standardized *G. biloba* extract (Gb) contains flavonoids and terpene trilactones, and possesses free radical-scavenging and antioxidant activities [26, 27]. Gb is commonly consumed as a dietary supplement for many disorders of the central nervous system (CNS), such as memory impairment, AD, and multi-infarct dementia [28–31]. Gb has been shown to protect against Aβ-induced neurotoxicity [32, 33]. In addition, Gb showed anti-inflammatory activity through the antagonism of PAF [34, 35]. Among the constituents of terpene trilactones, ginkgolides A, B, and C are highly selective and competitive PAFR antagonists [33].

The novel extract of *G. biloba*, YY-1224, consists of 24% flavonoids and 12% terpene trilactones (ginkgolides A, B, and C and bilobalide) (Table 1 and Additional file 1: Figure S1). Given that Gb generally contains 22–27% flavonoid glycosides and 5–7% terpene lactones, increased terpenoid levels are likely to be responsible for the protective activity of YY-1224 [36]. Bilobalide and ginkgolide B have neuroprotective activity [37–42]. Pilot studies indicated that YY-1224 had significant neurotrophic and antioxidative activities (Additional file 1: Figures S3 and S4). In addition, YY-1224 inhibited the COX-2 mRNA or protein expression induced by Aβ (1-42) in mouse hippocampus, PC12 cells, or mixed cortical cells (Additional file 1: Figure S5)*.* Furthermore, YY-1224 attenuated Aβ (1-42)-induced cell death in PC12 cells or mixed cortical cells (Additional file 1: Figure S6). We used COX-2 knockout (−/−) mice and APPswe/PS1dE9 transgenic (APP/PS1 Tg) mice to examine whether YY-1224 affects Aβ (1-42)-induced learning impairment and inflammatory responses when compared with Gb. Our results suggest that treatment with YY-1224 significantly attenuates Aβ (1-42)-induced memory impairments and pro-inflammatory responses via COX-2 suppression by inhibiting PAF and activating PPARγ. In addition, the prolonged treatment with YY-1224 enhances memory function and decreases Aβ peptide deposits and pro-inflammatory microglial activation in APP/PS1 Tg mice via COX-2 inhibition.

**Table 1:** Contents of Gb and YY-1224

Contents		Gb (%)	YY-1224 (%)
Ginkgo flavone glycosides	Quercetin	24	24.6
	Kaempferol		
	Isorhamnetin		
Terpene trilactones	Bilobalide	6	12.9
	Ginkgolide A		
	Ginkgolide B		
	Ginkgolide C		

## Methods

### Animals and drug treatment

The present study was performed in accordance with the Institute for Laboratory Research (ILAR) Guidelines for the Care and Use of Laboratory Animals. COX-2 (−/−)- and COX-2 (+/+)- mice were described previously [43, 44]. Breeding pairs of double transgenic mice expressing Swedish mutant amyloid precursor protein gene and mutant presenilin-1 (deletion of exon 9) gene [APPswe/PSEN1dE9 double Tg mice; B6C3-Tg (APPswe, PSEN1dE9) 85Dbo/J, The Jackson Laboratory, Bar Harbor, ME, USA] were bred and housed in an approved animal facility at Kangwon National University. Animals were maintained on a 12/12-h light/dark cycle and fed ad libitum and were adapted to these conditions for 2 weeks before the experiment.

Aβ (1-42) and Aβ (42-1) were purchased from American Peptide (Sunnyvale, CA, USA). Aβ (1-42) and Aβ (42-1) were dissolved in 0.1 M PBS at pH 7.4, and aliquots were stored at −20 °C. Aβ peptides in each aliquot were aggregated by incubation in sterile distilled water at 37 °C for 4 days. Six-month-old COX-2 (−/−)- and COX-2 (+/+)-mice were administered Aβ (1-42) and Aβ (42-1) (400 pmol, i.c.v. injection) according to the procedure established by Laursen and Belknap [45]. Each mouse was injected in the bregma using a 10-μl microsyringe (Hamilton, Reno, NV, USA) fitted with a 26-gauge needle inserted at a depth of 2.4 mm. The injection volume was 5 μl. The injection placement and needle track were visible and could be verified during dissection.

**Fig. 1:**
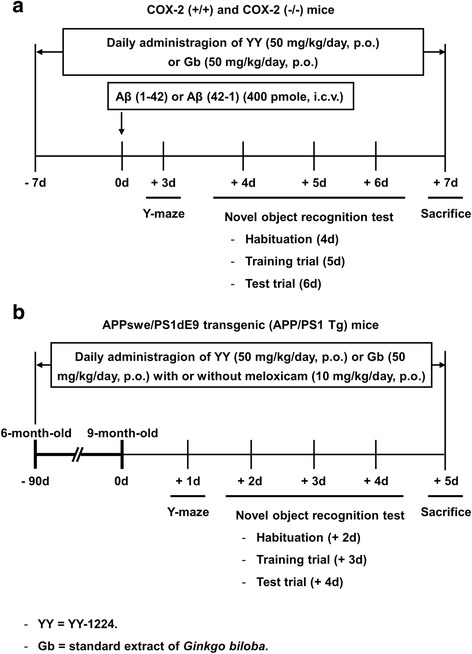
Experimental design for evaluating the effects of YY-1224 or Gb on learning impairments in mice. **a** Effects of YY-1224 or Gb on Aβ (1-42)-induced memory impairment in COX-2 (+/+) and COX-2 (−/−) mice (Figs. 2, 3, 4, 5, 6, 7, and 8). Mice received YY-1224 or Gb for 14 consecutive days [7 days before Aβ (1-42) i.c.v. infusion and 7 days period of memory assessment after Aβ (1-42) i.c.v. infusion]. **b** Effects of meloxicam on the pharmacological activity of YY-1224 or Gb in response to neurotoxic changes (Figs. 9, 10, 11, 12, and 13) in APP/PS1 Tg mice. Mice received YY-1224 or Gb with or without meloxicam 90 consecutive days and additional 5-day period of memory assessment

YY-1224 and a standard *G. biloba* extract (Gb) were obtained from the research center of Yuyu Pharma Inc. (Suwon, Republic of Korea). The content and representative HPLC chromatogram of each component in YY-1224 or Gb are shown in Table 1 and Additional file 1: Figure S1, respectively. YY-1224 (50 mg/kg) or Gb (50 mg/kg) was dissolved in 10% tween-80 and administered orally in a volume of 1 ml/kg. YY-1224 or Gb administration began 7 days before the Aβ i.c.v. injection, and the drug administration was continued once a day throughout the experimental period. The behavioral study commenced on day 3 after Aβ i.c.v. injection and was carried out sequentially. During the behavioral study, YY-1224 or Gb was administered 30 min after the behavioral test to avoid a direct effect on performance. The experimental design is shown in Fig. 1a and Additional file 1: Figure S2.

In addition, 6-month-old APPswe/PS1dE9 double Tg mice were treated with YY-1224 (50 mg/kg, p.o.) or Gb (50 mg/kg, p.o.) with or without the preferential COX-2 inhibitor, meloxicam (10 mg/kg, p.o.; Sigma-Aldrich, St. Louis, MO, USA), once a day for 3 months. Meloxicam was suspended in 0.5% sodium carboxymethyl cellulose (Na-CMC) immediately before use. The behavioral study was started when the mice were 9 months old, and additional treatment with YY-1224, Gb, or meloxicam was continued during the behavioral study. During the behavioral study, drugs were administered 30 min after the behavioral test to avoid a direct effect on performance. The experimental design is shown in Fig. 1b.

### Y-maze test

The Y-maze test was performed as described previously [46]. Briefly, the Y-shaped maze was constructed of black acrylic with three identical arms separated by 120°. Each arm was 40 cm long, 12 cm tall, and 10 cm wide. The mouse was placed at the end of one arm and allowed to move freely through the maze during an 8-min session. The percent alternation was calculated as the ratio of actual to possible alternations (defined as the total number of arm entries minus two) multiplied by 100.

### Novel object recognition test

The novel object recognition test was performed as described previously [47, 48]. On the training trial, two different objects were fixed on the floor in a symmetric position from the center of the open field box (40 × 40 × 35 high cm), 20 cm from each other and 10 cm from the nearest wall, and each mouse was allowed to explore the objects for 10 min. Mice were subjected to the test trial 24 h after the training trial. One of the familiar objects used during training trial was replaced by a novel object, and each mouse was then allowed to explore the objects for 10 min. Time spent exploring each object was recorded and analyzed with a video tracking system (EthoVision, Noldus, The Netherlands). Novel object recognition was expressed as a “recognition index (%),” the percentage of time spent exploring the novel object over the total time spent exploring both objects.

### Immunocytochemistry

Immunocytochemistry and staining quantification were performed as described previously [46, 49, 50]. Mice were perfused transcardially with 50 mL of ice-cold PBS (10 mL/10 g body weight) followed by 4% paraformaldehyde (20 mL/10 g body weight). Brains were removed and stored in 4% paraformaldehyde overnight. The brains were cut on a horizontal sliding microtome into 35-μm transverse free-floating sections. Every sixth section of the dorsal hippocampus (total of four sections) from each brain was collected for immunocytochemistry. Sections were blocked with PBS containing 0.3% hydrogen peroxide for 30 min and then incubated in PBS containing 0.4% Triton X-100 and 1% normal serum for 20 min. Antigen retrieval with formic acid (70%, 10 min) was performed to detect Aβ deposition in APPswe/PS1dE9 double Tg mice. After a 48-h incubation with primary antibody against PAFR (1:200; Cayman Chemical, Ann Arbor, MI, USA), PAF-AH (1:80; Santa Cruz Biotechnology, Inc., Santa Cruz, CA, USA), Aβ (1:50; Invitrogen, Thermo Fisher Scientific, Camarillo, CA, USA), or Iba-1 (1:500, Wako Pure Chemical Industries, Chuo-ku, Osaka, Japan), sections were incubated with the biotinylated secondary antibody (1:1000; Vector Laboratories, Burlingame, CA, USA) for 1 h. The sections were then immersed in a solution containing avidin–biotin peroxidase complex (Vector Laboratories) for 1 h, and 3,3′-diaminobenzidine was utilized as the chromogen. Digital images were acquired at ×4, ×10, or ×20 objective magnification using an Olympus microscope (BX51; Olympus) and a digital microscope camera (DP72; Olympus). ImageJ version 1.47 software (National Institutes of Health, Bethesda, MD, USA) was employed to quantify images. Briefly, images were converted to 8-bit grayscale images and were subjected to background subtraction to correct for uneven background. To measure PAFR- or PAF-AH-immunoreactivity, the pyramidal cell layer of CA1 and CA3 regions from each section were selected as the region of interest (ROI). Threshold values were set to select the immunoreactive area. The integrated density of each ROI was measured, and the value was expressed as density per micrometer square. For staining quantification of Aβ or Iba-1, the entire hippocampus and the cortical area containing primary somatosensory cortex were selected as the ROIs. Threshold values were set to identify immunoreactive areas, and particle analysis was employed to measure the area fraction of Aβ- or Iba-1-immunoreactivity (Additional file 1: Figure S13).

### Western blot

Tissues were lysed in buffer containing a 200-mM Tris–HCl (pH 6.8), 1% SDS, 5 mM EGTA, 5 mM EDTA, 10% glycerol, 1× phosphatase inhibitor cocktail I (Sigma-Aldrich), and 1× protease inhibitor cocktail (Sigma-Aldrich). The lysate was centrifuged at 12,000 × *g* for 30 min, and the supernatant fraction was used for Western blot analysis as described previously [48–50]. Proteins (20 μg/lane) were separated by 8 or 10% sodium dodecyl sulfate-polyacrylamide gel electrophoresis (PAGE) and transferred onto polyvinylidene fluoride (PVDF) membranes. Following transfer, the membranes were preincubated with 5% non-fat milk for 30 min and incubated overnight at 4 °C with primary antibody against PPAR-γ (1:200; Santa Cruz Biotechnology, Inc.) or β-actin (1:50000; Sigma-Aldrich). Membranes were then incubated with HRP-conjugated secondary anti-rabbit IgG (1:1000, GE Healthcare, Piscataway, NJ, USA) or anti-mouse IgG (1:1000, Sigma-Aldrich) for 2 h. Subsequent visualization was performed using an enhanced chemiluminescence system (ECL plus^®^, GE Healthcare). Relative intensities of the bands were quantified with PhotoCapt MW (version 10.01 for Windows; Vilber Lourmat, Marne la Vallée, France), and then normalized to the intensity of β-actin.

### Reverse transcription and real-time polymerase chain reaction (RT-rt-PCR)

Reverse transcription and real-time polymerase chain reaction (RT-rt-PCR) was performed as described previously [51]. Total RNA was isolated from the hippocampus using an RNeasy Mini Kit (Qiagen, Valencia, CA, USA) according to the manufacturer’s instructions. Reverse transcription reactions were carried out using the RNA to cDNA EcoDry Premix (Clontech, Palo Alto, CA, USA) with a 1-h incubation at 42 °C. For rt-PCR, 0.5 μL of complementary DNA (cDNA) was added to 50 μL of total PCR reaction mixture, containing 25 pmol of each primer and QuantiTect SYBR Green PCR Master Mix (Qiagen). rt-PCR was performed using a CFX96 Touch real-time PCR system (Bio-Rad Laboratories, Hercules, CA, USA). The reference gene (GAPDH) and target gene from each sample were run in parallel in the same plate with the same amount of cDNA. Primer sequences are listed in Table 2. After activation of HotStarTaq DNA polymerase at 95 °C for 15 min, PCR was performed as 40 cycles of denaturation at 94 °C for 1 min, annealing at 60 °C for 1 min, and extension at 72 °C for 1 min. The relative mRNA expression level was normalized to that of GAPDH (2^[Ct(GAPDH)-Ct(each gene)]^).

**Table 2:** Gene primer sequences for RT-rt-PCR analysis

Gene	Primer sequences (5′-3′)	Expected size (bp)	Gene bank accession number
PAFR	F: CAACGAGGGCGACTGGATT	97	D50872.1 [93]
	R: GACACCCAAAAAGGCCACACT		
PAF-AH I α1	F: ACACAGCATGTACTCTGGCG	293	BC067015.1 [93]
	R: GCATCTAAGAAGTGGGCTCG		
PAF-AH I α2	F: AGAATGCCAAGGTGAACCAG	127	BC056211.2 [94]
	R: AAATCAAACATGTCGTGCCA		
PAF-AH I LIS1	F: GATGACAAGACCCTCCGTGT	240	NM_013625.4 [95]
	R: GAGCTCAAATGGGGTAACCA		
PAF-AH II	F: ATCAAGGAAGGGGAGAAGGA	200	BC021890.1 [96]
	R: AAGGAGTGACCCATCACGGC		
TNF-α	F: CATCTTCTCAAAATTCGAGTGACAA	175	D84199.2 [97]
	R: TGGGAGTAGACAAGGTACAACCC		
IL-1β	F: GAAAGACGGCACACCCACC	83	BC011437.1 [98]
	R: AGACAAACCGCTTTTCCATCTTC		
IL-6	F: TGGAGTCACAGAAGGAGTGGCTAAG	155	BC132458.1
	R: TCTGACCACAGTGAGGAATGTCCAG		
INF-γ	F: TCAAGTGGCATAGATGTGGAAGAA	92	BC119063.1 [97]
	R: TGGCTCTGCAGGATTTTCATG		
iNOS	F: CAGCTGGGCTGTACAAACCTT	95	BC062378.1 [97]
	R: CATTGGAAGTGAAGCGTTTCG		
PPAR-α	F: CCCTGAACATCGAGTGTCGAA	142	BC016892.1 [99]
	R: TTGCAGCTCCGATCACACTT		
PPAR- γ	F: TGTCGGTTTCAGAAGTGCCTT	146	AB644275.1
	R: GCTCGCAGATCAGCAGACTCT		
YM1	F: ACCCCTGCCTGTGTACTCACCT	183	BC061154.1 [100]
	R: CACTGAACGGGGCAGGTCCAAA		
CD206	F: TCTTTGCCTTTCCCAGTCTCC	241	NM_008625.2 [101]
	R: TGACACCCAGCGGAATTTC		
CD16	F: TTTGGACACCCAGATGTTTCAG	163	NM_010188.5 [101]
	R: GTCTTCCTTGAGCACCTGGATC		
CD32	F: AATCCTGCCGTTCCTACTGATC	187	BC038070.1 [101]
	R: GTGTCACCGTGTCTTCCTTGAG		
CD86	F: TTGTGTGTGTTCTGGAAACGGAG	202	BC013807.1 [101]
	R: AACTTAGAGGCTGTGTTGCTGGG		
COX-2	F: CCACTTCAAGGGAGTCTGGA	197	NM_011198.3 [102]
	R: AGTCATCTGCTACGGGAGGA		
GAPDH	F: GCCAAGGCTGTGGGCAAGGT	112	GU214026.1 [103]
	R: TCTCCAGGCGGCACGTCAGA		

### Determination of malondialdehyde

The amount of lipid peroxidation in the hippocampus was determined by measuring the level of thiobarbituric acid-reactive substance in homogenates and is expressed in terms of malondialdehyde (MDA) content. The MDA level was measured using HPLC-UV/VIS detection system (model LC-20AT and SPD-20A, Shimadzu, Kyoto, Japan) as described by Richard et al. [52] with slight modifications [49, 53]. Briefly, each hippocampal tissue was homogenized in PBS, and 0.1 ml of this homogenate (or standard solutions prepared daily from 1,1,3,3-tetra-methoxypropane) and 0.75 ml of the working solution (thiobarbituric acid 0.37% and perchloric acid 6.4%, 2:1, *v*:*v*) were mixed and heated to 95 °C for 1 h. After cooling (10 min in ice water bath), the flocculent precipitate was removed by centrifugation at 3200 × *g* for 10 min. The supernatant was neutralized and filtered prior to injection. Isocratic separation was achieved on a 5-μm ODS column with mobile phase consisting of 50 mM PBS (pH 6.0): methanol (58:42, *v*/*v*) at a flow rate of 1.0 mL/min. The effluents were monitored at 532 nm.

### Determination of protein carbonyls

The extent of protein oxidation in the hippocampus was assessed by measuring the content of protein carbonyl groups, which was determined spectrophotometrically with the 2,4-dinitrophenylhydrazine (DNPH)-labeling procedure, as described by Oliver et al. [54]. Results are expressed as nanomoles of DNPH incorporated/mg protein [49, 53] based on the extinction coefficient for aliphatic hydrazones at 21 mM^−1^·cm^−1^.

### Determination of synaptosomal reactive oxygen species (ROS) formation

Synaptosomal fractions were obtained as described by Whittaker et al. [55], with minor modifications [49, 53]. The protein concentration of the synaptosomal preparation was measured using the BCA protein assay reagent (Pierce, Rockford, IL, USA) and was found to be approximately 5 mg of protein per milliliter. ROS formation was assessed by measuring the conversion from 2′,7′-dichlorofluorescin diacetate (DCFH-DA) to dichlorofluorescin (DCF) as described by Lebel and Bondy [56]. Briefly, hippocampal synaptosomes were incubated with 5 μM DCFH-DA (Molecular Probes, Eugene, OR, USA) for 15 min at 37 °C. Excess unbound probe was removed by centrifugation at 12,500 × *g* for 10 min. The fluorescence intensity due to ROS was measured at an excitation wavelength of 488 nm and an emission wavelength of 528 nm. DCF (Sigma-Aldrich) was used as a standard.

### Determination of 4-hydroxy-2-nonenal (4-HNE) and protein carbonyl by slot blot analysis

Determination of 4-HNE was performed using slot blot analysis as described previously [49]. Following adsorption, the PVDF membranes were preincubated with 5% non-fat milk and incubated overnight at 4 °C with anti-4-HNE antibody (1:2000, Calbiochem, La Jolla, CA, USA). After incubation with the primary antibody, membranes were incubated with a HRP-conjugated secondary antibody. Subsequent visualization was performed using an enhanced chemiluminescence system (ECL plus®, GE Healthcare). The amount of oxidized proteins was measured using the Oxyblot kit [Chemicon (EMD Millipore), Temecula, MA, USA] according to the instructions provided by the manufacturer. Briefly, the protein carbonyl content was labeled with protein hydrazone derivatives using 2,4-dinitrophenylhydrazide (DNP). Each blot was preincubated with 5% non-fat milk and then incubated with the primary antibody (1:100) specific to the DNP moiety, followed by incubation with a HRP-conjugated secondary antibody. Subsequent visualization was performed using an enhanced chemiluminescence system (ECL plus®, GE Healthcare) [49].

### Statistics

Data were analyzed using IBM SPSS ver. 21.0 (IBM, Chicago, IL, USA). Analysis of variance (ANOVA) was employed for the statistical analysis of the effect of Aβ treatment, pretreatment (YY-1224 or Gb), or COX-2 inhibition (COX-2 gene knockout or COX-2 inhibitor). Post hoc Fisher’s least significant difference pairwise comparisons tests were then conducted*. P* values <0.05 were considered to be significant.

## Results

### Effect of YY-1224 or Gb on Aβ (1-42)-induced impairment of visual recognition learning in COX-2 (+/+)- and COX-2 (−/−)-mice

**Fig. 2:**
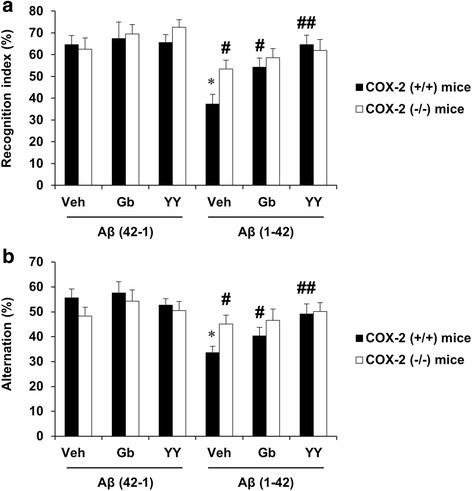
Effects of YY-1224 (YY) or Gb on Aβ (1-42)-induced memory impairment in mice. **a** Novel object recognition test. **b** Y-maze test. *Veh* vehicle for YY or Gb (10% tween-80 in sterile saline). Each value is the mean ± S.E.M of six animals. *P < 0.01 vs. COX-2 (+/+) mice treated with vehicle + Aβ (42-1); ^#^ *P* < 0.05, ^##^ *P* < 0.01 vs. COX-2 (+/+) mice treated with vehicle + Aβ (1-42) (three-way ANOVA was followed by Fisher’s LSD pairwise comparisons)

The recognition test is based on the natural tendency of rodents to investigate a novel object instead of a familiar one. The choice to explore the novel object reflects the use of learning and (recognition) memory processes. There was no significant difference between Aβ-treated groups in exploratory preference for objects in the training trial. The exploratory preference for a novel object was significantly decreased [Fig. 2a, ANOVA and post hoc pairwise comparisons showing the effect of Aβ (1-42), *P* < 0.01] in Aβ (1-42)-treated mice when compared with Aβ (42-1)-treated mice. In the COX-2 (+/+) mice, repeated treatment with Gb or YY-1224 significantly ameliorated the decrease in exploratory preference for a novel object that was induced by Aβ (1-42) [Fig. 2a, ANOVA and post hoc pairwise comparisons indicating the effect of Gb (*P* < 0.05) or YY-1224 (*P* < 0.01)]. Genetic depletion of COX-2 also significantly attenuated the Aβ (1-42)-induced decrease in the exploratory preference for a novel object (Fig. 2a, ANOVA and post hoc pairwise comparisons showing the effect of COX-2 gene knockout, *P* < 0.05), and YY-1224 or Gb did not provide additional cognitive enhancing effects in COX-2 (−/−) mice.

### Effects of YY-1224 or Gb on Aβ (1-42)-induced impairment of spatial working memory in COX-2 (+/+)- and COX-2 (−/−)-mice

The Y-maze test is based on the natural navigation behaviors of rodents and is used to evaluate spatial working memory, which is known to be dependent on hippocampal function. There was no significant change in the performance of the Y maze task between Aβ (42-1)-treated groups. In the COX-2 (+/+) mice, alternation behavior was significantly decreased [Fig. 2b, ANOVA and post hoc pairwise comparisons showing the effect of Aβ (1-42), *P* < 0.01] in Aβ (1-42)-treated mice compared to Aβ (42-1)-treated mice. ANOVA and post hoc pairwise comparisons revealed that the Aβ (1-42)-induced decrease in alternation behavior was significantly attenuated by YY-1224 (Fig. 2b, *P* < 0.01), Gb (Fig. 2b, *P* < 0.05), or genetic inhibition of COX-2 (Fig. 2b, *P* < 0.05). YY-1224-mediated attenuation appeared to be more pronounced than Gb in COX-2 (+/+) mice; however, YY-1224 and Gb did not affect the Y-maze performance in Aβ (1-42)-treated COX-2 (−/−) mice. The results from the Morris water maze are comparable to those from the novel object recognition test and the Y-maze test (Additional file 1: Figure S7).

### Effects of YY-1224 or Gb on Aβ (1-42)-induced changes in the expressions of PAFR and PAF-AH in the hippocampi of COX-2 (+/+)- and COX-2 (−/−)-mice

**Fig. 3:**
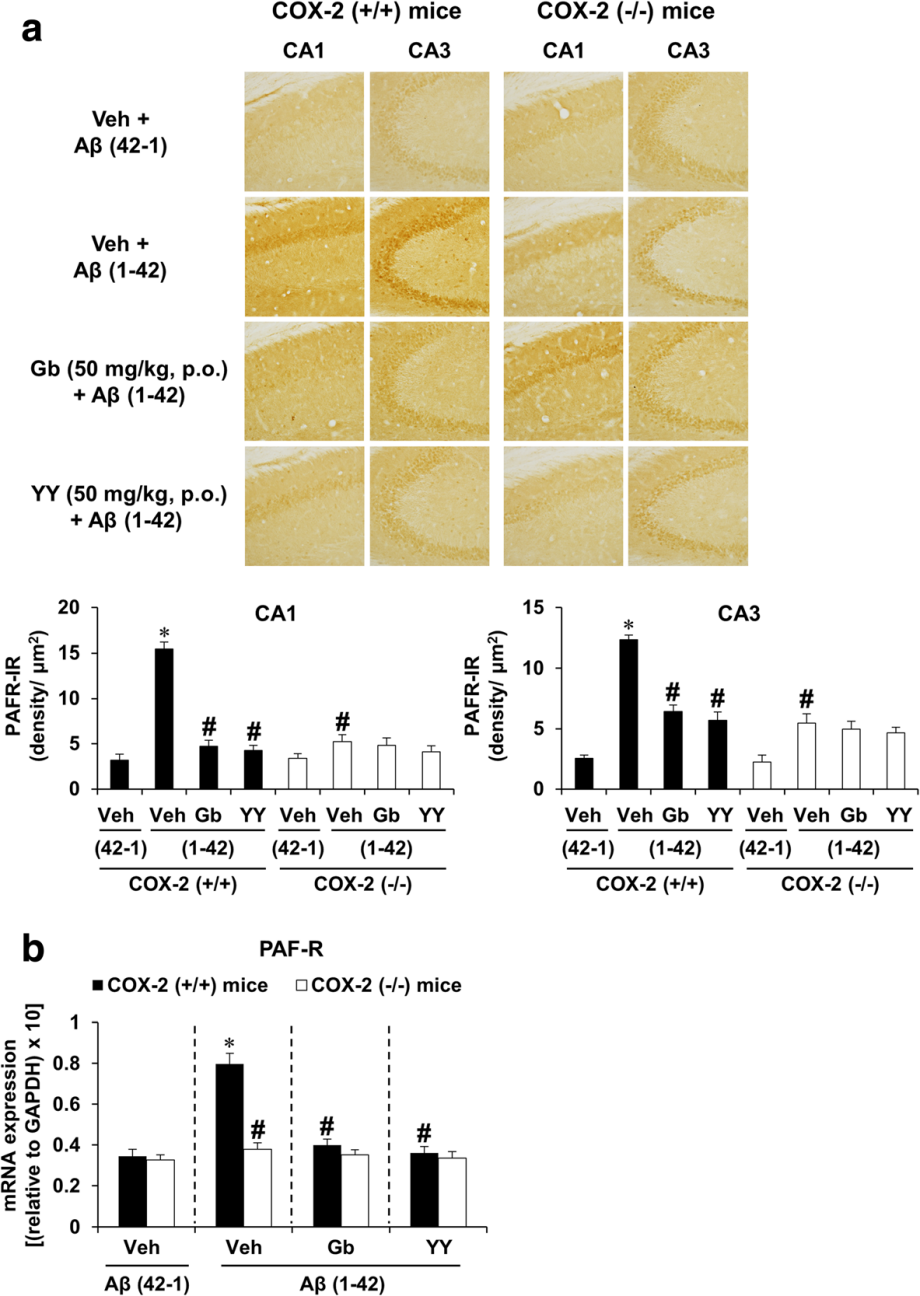
Effects of YY-1224 (YY) or Gb on Aβ (1-42)-induced PAFR expression in the hippocampi of mice. **a** Effect of YY on PAFR-immunoreactivity. **b** Effect of YY on PAFR mRNA expression. *Veh* vehicle for YY or Gb (10% tween-80 in sterile saline). Each value is the mean ± S.E.M of six animals. *P < 0.01 vs. COX-2 (+/+) mice treated with vehicle + Aβ (42-1); ^#^ *P* < 0.01 vs. COX-2 (+/+) mice treated with vehicle + Aβ (1-42) (three-way ANOVA was followed by Fisher’s LSD pairwise comparisons)

To explore whether PAF modulatory actions were involved in the pharmacological effect of YY-1224 against Aβ (1-42)-induced memory impairments, the expressions of PAFR and PAF-AH were examined in the hippocampus of COX-2 (+/+)- and COX-2 (−/−)-mice using immunocytochemistry and RT-rt-PCR. ANOVA and post hoc pairwise comparisons indicated that Aβ (1-42) infusion significantly increased PAFR-immunoreactivity (Fig. 3a, *P* < 0.01 for CA1 or CA3) and PAFR mRNA levels (Fig. 3b, *P* < 0.01) in the hippocampi of COX-2 (+/+) mice. Aβ (1-42)-induced increases in PAFR-immunoreactivity and PAFR mRNA expression were significantly attenuated by Gb, YY-1224, or COX-2 gene knockout (Fig. 3a, b, ANOVA and post hoc pairwise comparisons showing the effect of Gb, YY-1224, or COX-2 gene knockout, *P* < 0.01). In addition, YY-1224 and Gb did not show additional effects compared to COX-2 gene depletion (Fig. 3). The results from RT-rt-PCR of PAFR mRNA are comparable to those from RT-PCR (Additional file 1: Figure S8a).

**Fig. 4:**
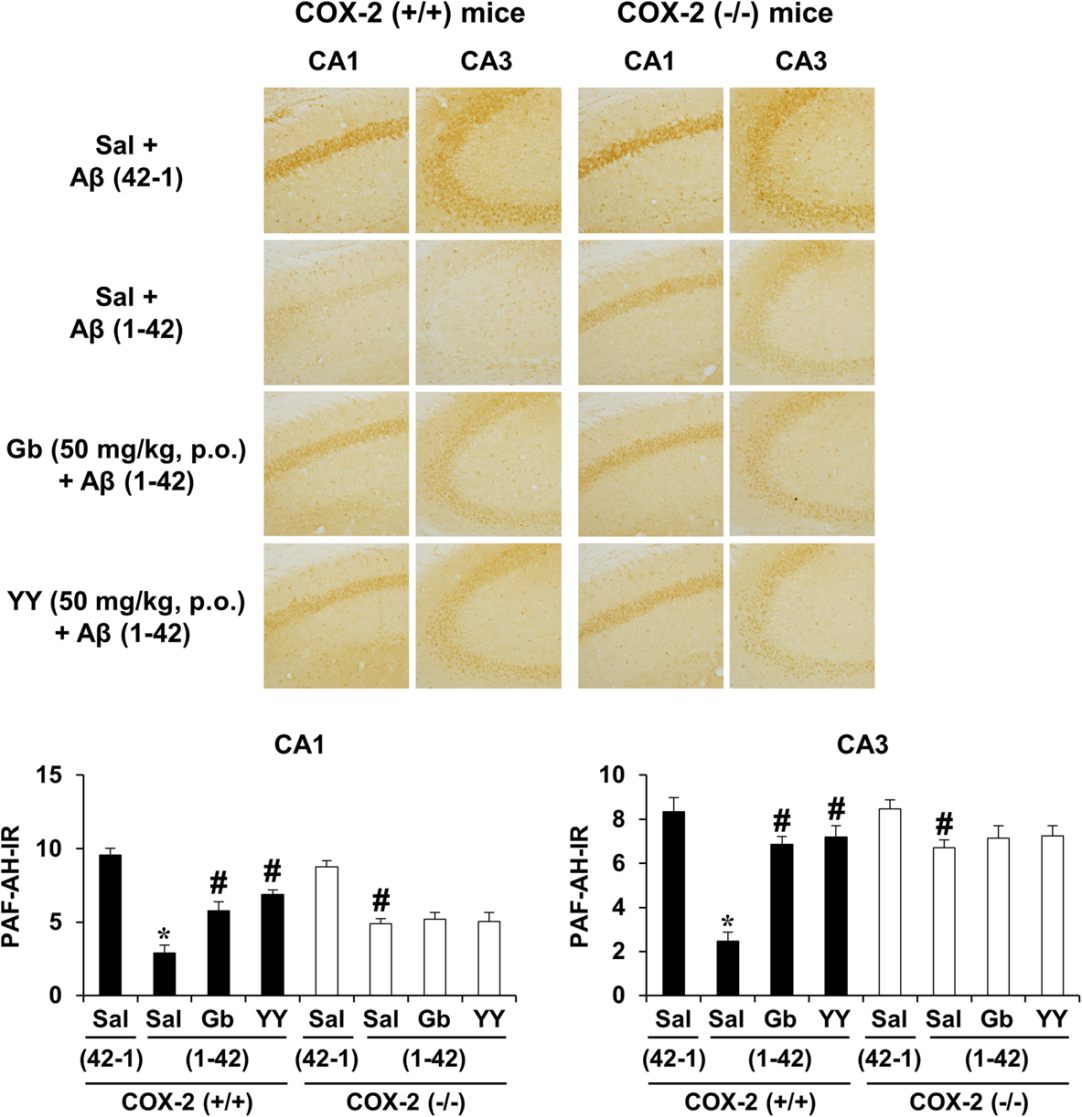
Effects of YY-1224 (YY) or Gb on Aβ (1-42)-induced PAF-AH I-immunoreactivity in the hippocampi of mice. *Veh* vehicle for YY or Gb (10% tween-80 in sterile saline). Each value is the mean ± S.E.M of six animals. *P < 0.01 vs. COX-2 (+/+) mice treated with vehicle + Aβ (42-1); ^#^ *P* < 0.01 vs. COX-2 (+/+) mice treated with vehicle + Aβ (1-42) (three-way ANOVA was followed by Fisher’s LSD pairwise comparisons)

**Fig. 5:**
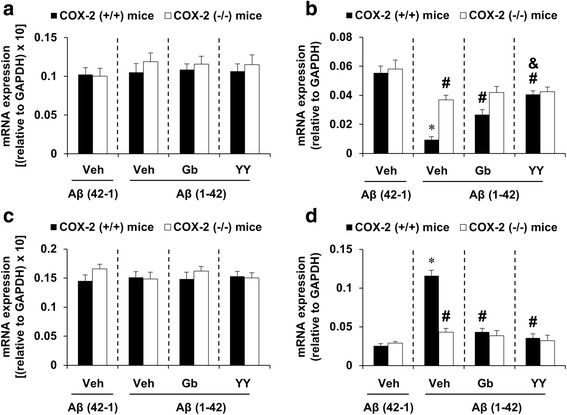
Effects of YY-1224 (YY) or Gb on Aβ (1-42)-induced PAF-AH mRNA levels in the hippocampus. **a** Changes in PAF-AH I α1 mRNA expression. **b** Changes in PAF-AH I α2 mRNA expression. **c** Changes in PAF-AH I LIS1 mRNA expression. **d** Changes in PAF-AH II mRNA expression. *Veh* vehicle for YY or Gb (10% tween-80 in sterile saline). Each value is the mean ± S.E.M of six animals. *P < 0.01 vs. COX-2 (+/+) mice treated with vehicle + Aβ (42-1); ^#^ *P* < 0.01 vs. COX-2 (+/+) mice treated with vehicle + Aβ (1-42); &*P* < 0.05 vs. COX-2 (+/+) mice treated with Gb + Aβ (1-42) (three-way ANOVA was followed by Fisher’s LSD pairwise comparisons)

In contrast, total PAF-AH I-immunoreactivity was significantly decreased by Aβ (1-42) in the hippocampi of COX-2 (+/+) mice [Fig. 4, ANOVA and post hoc pairwise comparisons showing the effect of Aβ (1-42), *P* < 0.01]. To examine which subunit of PAF-AH is transcriptionally regulated by Aβ (1-42), we examined changes in the mRNA expression level of PAF-AH I subunits using RT-rt-PCR. Aβ (1-42) infusion significantly decreased the mRNA expression of the α2 subunit, but not the α1 or LIS1 subunit, in the hippocampus of COX-2 (+/+) mice [Fig. 5b, ANOVA and post hoc pairwise comparisons showing the effect of Aβ (1-42), *P* < 0.01]. In addition, the PAF-AH II mRNA level was significantly increased after treatment with Aβ (1-42) [Fig. 5d, ANOVA and post hoc pairwise comparisons showing the effect of Aβ (1-42), *P* < 0.01]. These changes in PAF-AH I-immunoreactivity, PAF-AH I α2 mRNA expression, and PAF-AH II mRNA expression were significantly reversed by Gb, YY-1224, and COX-2 gene depletion (Fig. 5b, d, ANOVA and post hoc pairwise comparisons showing the effect of Gb, YY-1224, or COX-2 gene knockout, *P* < 0.01). YY-1224 appeared to be more effective in enhancing PAF-AH 1α2 mRNA expression than Gb in COX-2 (+/+) mice (Fig. 5b, ANOVA and post hoc pairwise comparisons showing the difference between Gb and YY-1224, *P* < 0.05). YY-1224 and Gb did not show additional effects compared to COX-2 gene depletion in the presence of Aβ (1-42) (Figs. 4 and 5). The results from RT-rt-PCR of the mRNA expression of PAF-AH subtypes are comparable to those from RT-PCR (Additional file 1: Figure S8b-d).

### Effects of YY-1224 or Gb on lipid peroxidation, protein oxidation, and reactive oxygen species (ROS) formation induced by Aβ (1-42) in the hippocampi of COX-2 (+/+)- and COX-2 (−/−)-mice

**Fig. 6:**
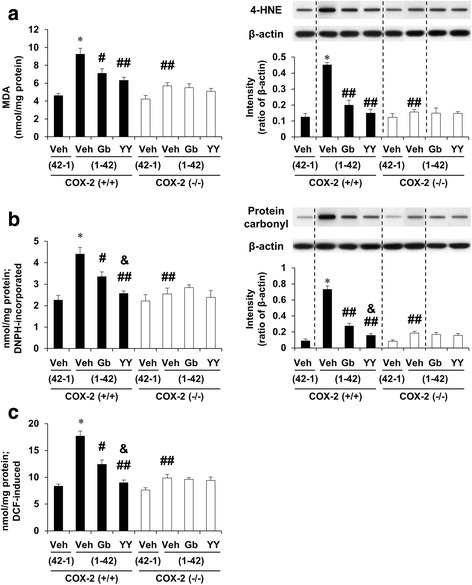
Effects YY-1224 (YY) or Gb on Aβ (1-42)-induced oxidative burdens in the hippocampus of mice. **a** Aβ (1-42)-induced lipid peroxidation. **b** Aβ (1-42)-induced protein oxidation. Lipid peroxidation and protein oxidation were evaluated by quantitative biochemical analyses and slot blot analyses. **c** Aβ (1-42)-induced synaptosomal formation of reactive oxygen species. *Veh* vehicle for YY or Gb (10% tween-80 in sterile saline). Each value is the mean ± S.E.M of six animals. *P < 0.01 vs. COX-2 (+/+) mice treated with vehicle + Aβ (42-1); ^#^ *P* < 0.05, ^##^ *P* < 0.01 vs. COX-2 (+/+) mice treated with vehicle + Aβ (1-42); &*P* < 0.05 vs. COX-2 (+/+) mice treated with Gb + Aβ (1-42) (three-way ANOVA was followed by Fisher’s LSD pairwise comparisons)

To evaluate whether COX-2 inhibitory action is involved in the pharmacological effect of YY-1224 against Aβ (1-42)-induced oxidative burdens, the level of oxidative stress markers, including lipid peroxidation, protein expression, and ROS formation was measured in the hippocampus of COX-2 (+/+)- and COX-2 (−/−)-mice. Treatment with Aβ (1-42) resulted in an increase in lipid peroxidation, as assessed by MDA and 4-HNE level, protein oxidation, as shown by protein carbonyl level and expression, and synaptosomal ROS formation in COX-2 (+/+) mice [Fig. 6, ANOVA and post hoc pairwise comparisons showing the effect of Aβ (1-42), *P* < 0.01]. ANOVA and post hoc pairwise comparisons indicated that YY-1224 and Gb treatment significantly attenuated Aβ (1-42)-induced lipid peroxidation (Fig. 6a, YY-1224 and Gb, *P* < 0.01 for MDA and 4-HNE), protein oxidation (Fig. 6b, YY-1224, *P* < 0.01 or Gb *P* < 0.05 for protein carbonyl level; YY-1224 and Gb, *P* < 0.01 for protein carbonyl expression), and synaptosomal ROS formation (Fig. 6c, YY-1224, *P* < 0.01 or Gb *P* < 0.05) in COX-2 (+/+) mice. COX-2 gene depletion also significantly attenuated oxidative stress induced by Aβ (1-42) (Fig. 6, ANOVA and post hoc pairwise comparisons showing the effect of COX-2 gene knockout, *P* < 0.01 for all parameters). YY-1224 attenuated Aβ (1-42)-induced protein oxidation and ROS formation in COX-2 (+/+) mice more effectively than Gb (Fig. 6b, c, ANOVA and post hoc pairwise comparisons showing the difference between Gb and YY-1224, *P* < 0.05), but neither showed additional effects over COX-2 gene depletion (Fig. 6).

### Effects of YY-1224 or Gb on changes in the expression of pro-inflammatory cytokines [tumor necrosis factor alpha (TNF-α), interleukin-1 beta (IL-1β), interleukin-6 (IL-6), and interferon gamma (IFN-γ)] and iNOS induced by Aβ (1-42) in the hippocampi of COX-2 (+/+)- and COX-2 (−/−)-mice

**Fig. 7:**
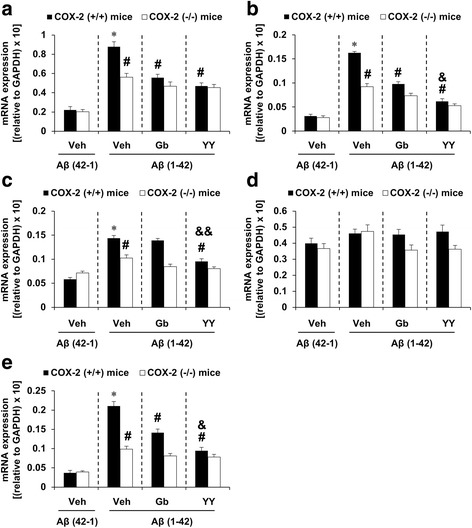
Effects of YY-1224 (YY) or Gb on Aβ (1-42)-induced proinflammatory genes in the hippocampi of mice. **a** Changes in TNF-α mRNA expression. **b** Changes in IL-1β mRNA expression. **c** Changes in IL-6 mRNA expression. **d** Changes in IFN-γ mRNA expression. **e** Changes in iNOS mRNA expression. *Veh* vehicle for YY or Gb (10% tween-80 in sterile saline). Each value is the mean ± S.E.M of six animals. *P < 0.01 vs. COX-2 (+/+) mice treated with vehicle + Aβ (42-1); ^#^ *P* < 0.01 vs. COX-2 (+/+) mice treated with vehicle + Aβ (1-42); &*P* < 0.05, &&*P* < 0.05 vs. COX-2 (+/+) mice treated with Gb + Aβ (1-42) (three-way ANOVA was followed by Fisher’s LSD pairwise comparisons)

To understand whether COX-2 inhibitory action was involved in the pharmacological effect of YY-1224 against Aβ (1-42)-induced pro-inflammatory changes, the mRNA levels of pro-inflammatory markers were examined in the hippocampus of COX-2 (+/+)- and COX-2 (−/−)-mice using RT-rt-PCR. As shown in Fig. 7, the mRNA expression of TNF-α, IL-1β, IL-6, and iNOS, but not IFN-γ, was significantly increased by Aβ (1-42) in COX-2 (+/+) mice [Fig. 7a–c, e, ANOVA and post hoc pairwise comparisons showing the effect of Aβ (1-42), *P* < 0.01]. YY-1224, Gb, and COX-2 gene depletion significantly attenuated these increases (Fig. 7a–c, e, ANOVA and post hoc pairwise comparisons showing the inhibitory effect of YY-1224, Gb, and COX-2 gene knockout, *P* < 0.01). ANOVA and post hoc pairwise comparisons showed that YY-1224 had a more pronounced effect than Gb on the increases in IL-1β, IL-6, and iNOS induced by Aβ (1-42) in COX-2 (+/+) mice (Fig. 7b, c and e, *P* < 0.05 for IL-1β and iNOS; *P* < 0.01 for IL-6). YY-1224 and Gb did not show additional effects compared to COX-2 gene depletion in the presence of Aβ (1-42) (Fig. 7). The results from RT-rt-PCR of the mRNA expression of pro-inflammatory factors were comparable to those from RT-PCR (Additional file 1: Figure S9).

### Effects of YY-1224 or Gb on changes in PPARγ expression induced by Aβ (1-42) in the hippocampi of COX-2 (+/+) and COX-2 (−/−) mice

**Fig. 8:**
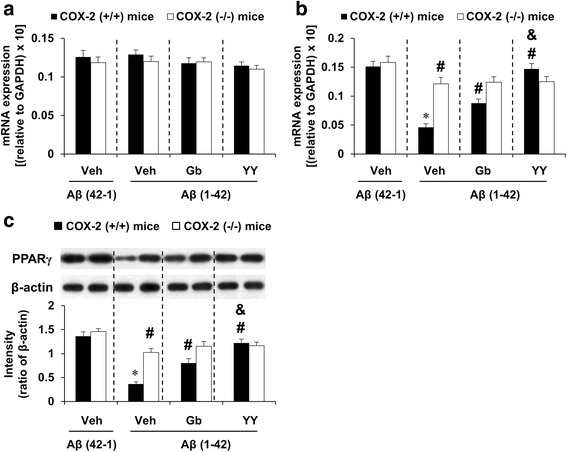
Effects of YY-1224 (YY) or Gb on Aβ (1-42)-induced PPAR expressions in the hippocampi of mice. **a** Changes in PPARα mRNA expression. **b** Changes in PPARγ mRNA expression. **c** Changes in PPARγ protein expression. *Veh* vehicle for YY or Gb (10% tween-80 in sterile saline). Each value is the mean ± S.E.M of six animals. *P < 0.01 vs. COX-2 (+/+) mice treated with vehicle + Aβ (42-1); ^#^ *P* < 0.01 vs. COX-2 (+/+) mice treated with vehicle + Aβ (1-42); &*P* < 0.05 vs. COX-2 (+/+) mice treated with Gb + Aβ (1-42) (three-way ANOVA was followed by Fisher’s LSD pairwise comparisons)

As mentioned above, it has been suggested that PPARγ is related to inflammatory and neurodegenerative processes [22]. To explore whether PPARs modulatory actions are involved in the anti-inflammatory effect of YY-1224, the mRNA or protein expression of PPARα or PPARγ was examined after Aβ (1-42) infusion in the hippocampus of COX-2 (+/+)- and COX-2 (−/−)-mice. Aβ (1-42) infusion significantly decreased the mRNA and protein expression of PPARγ in COX-2 (+/+) mice [Fig. 8b, c, ANOVA and post hoc pairwise comparisons showing the effect of Aβ (1-42), *P* < 0.01]. Aβ (1-42)-induced decreases in PPARγ mRNA and protein expression were significantly attenuated by YY-1224, Gb, or COX-2 gene knockout (Fig. 8b, c, ANOVA and post hoc pairwise comparisons showing the inhibitory effect of YY-1224, Gb, and COX-2 gene knockout, *P* < 0.01). YY-1224 mediated significant attenuation than Gb in COX-2 (+/+) mice (Fig. 8b, c, ANOVA and post hoc pairwise comparisons showing the difference between Gb and YY-1224, *P* < 0.05). YY-1224 and Gb did not show additional effects compared to COX-2 gene depletion (Fig. 8). The results from RT-rt-PCR of the PPARα or PPARγ mRNA expression were comparable to those from RT-PCR (Additional file 1: Figure S10).

### Effects of meloxicam, a preferential COX-2 inhibitor, on the pharmacological effect of YY-1224 on memory dysfunction and Aβ plaque deposition in APP/PS1 Tg mice

**Fig. 9:**
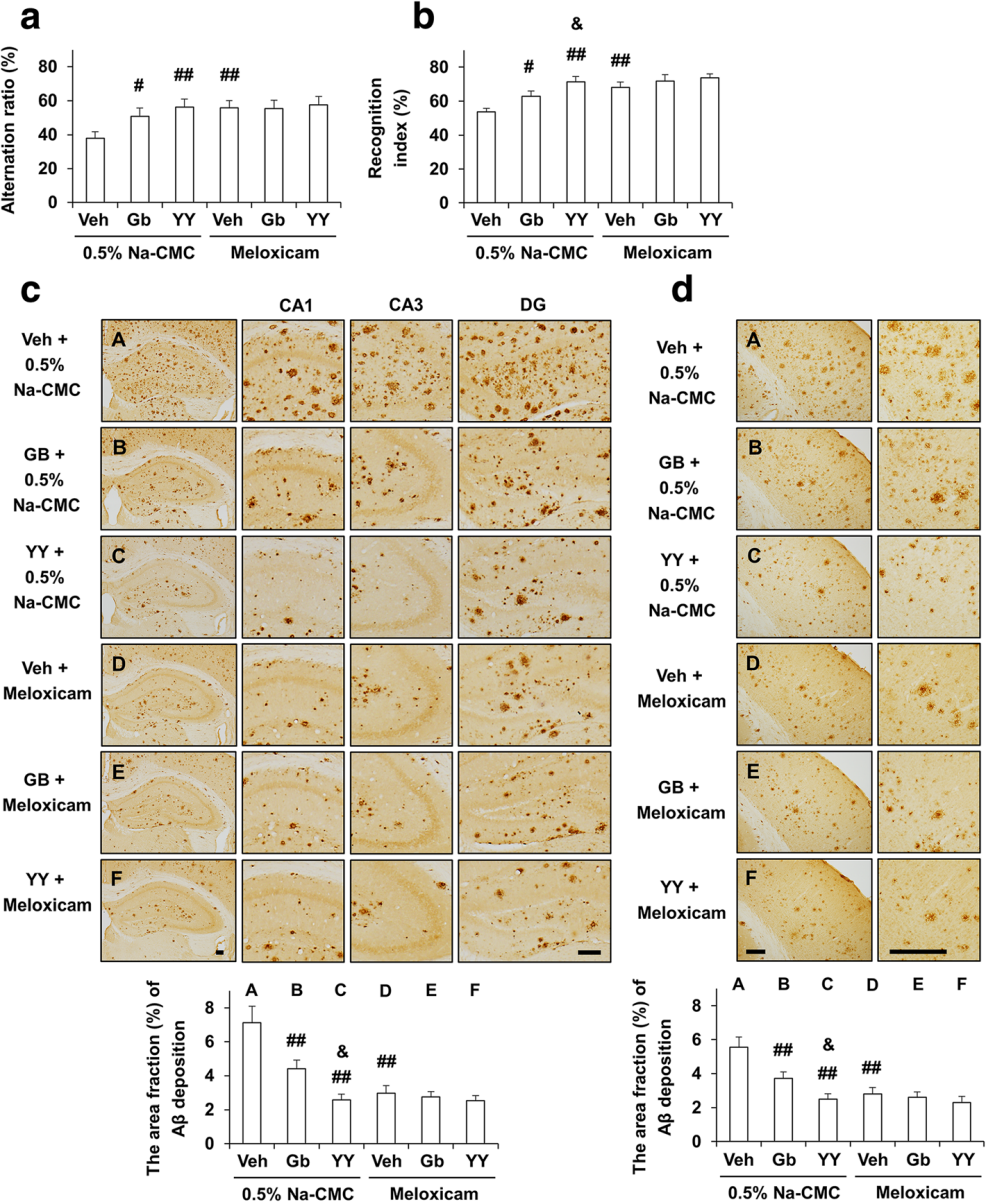
Activity of YY-1224 (YY) or Gb on memory impairment and Aβ deposition in APP/PS1 Tg mice. **a** Effects of meloxicam on the pharmacological activity of YY or Gb in response to Y-maze performance and **b** novel object recognition. **c**, **d** Effect of meloxicam on the pharmacological activity of YY or Gb in response to Aβ-immunoreactivity in the hippocampus (**c**) and cortex (**d**). *Veh* vehicle for YY or Gb (10% tween-80 in sterile saline). Each value is the mean ± S.E.M of 10 (**a**, **b**) or 5 (**c**, **d**) animals. ^#^ *P* < 0.05, ^##^ *P* < 0.01 vs. vehicle + 0.5% Na-CMC; & *P* < 0.05 vs. Gb + 0.5% Na-CMC (two-way ANOVA was followed by Fisher’s LSD pairwise comparisons). *Scale bar* = 200 μm

To understand the pharmacological effects of YY-1224, we examined the effect of Gb or YY-1224 on memory dysfunction and Aβ plaque deposition in APP/PS1 Tg mice. ANOVA and post hoc pairwise comparisons showed that treatment with YY-1224 or Gb significantly increased the alternation ratio in the Y-maze test (Fig. 9a, YY-1224 or Gb, *P* < 0.01 or *P* < 0.05) and the recognition index in the novel object recognition test (Fig. 9b, YY-1224 or Gb, *P* < 0.01 or *P* < 0.05). A preferential COX-2 inhibitor, meloxicam, also significantly enhanced cognitive function in the Y-maze (Fig. 9a *, P* < 0.01) and novel object recognition tests (Fig. 9b, *P* < 0.01). Consistent with results obtained from Aβ-treated mice, YY-1224 was more effective at enhancing novel object recognition in APP/PS1 Tg mice (Fig. 9b, ANOVA and post hoc pairwise comparisons showing the difference between Gb and YY-1224, *P* < 0.05), and meloxicam treatment did not affect the performance of YY-1224- or Gb-treated APP/PS1 Tg mice (Fig. 9a, b).

YY-1224, Gb, or meloxicam treatment significantly attenuated Aβ plaque deposition in the hippocampus and cortex of APP/PS1 Tg mice (Fig. 9c, d, ANOVA and post hoc pairwise comparisons showing the inhibitory effect of YY-1224, Gb, and meloxicam, *P* < 0.01 for hippocampus and cortex). Attenuation mediated by YY-1224 was more pronounced than attenuation mediated by Gb (Fig. 9c, d, ANOVA and post hoc pairwise comparisons showing the difference between Gb and YY-1224, *P* < 0.05 for hippocampus and cortex). Meloxicam did not affect responses to pharmacological activity in YY-1224- or Gb-treated APP/PS1 Tg mice (Fig. 9c, d).

### Effects of meloxicam on pharmacological activity mediated by YY-1224 through PAF-AH I and PPARγ expression in the hippocampi of APP/PS1 Tg mice

**Fig. 10:**
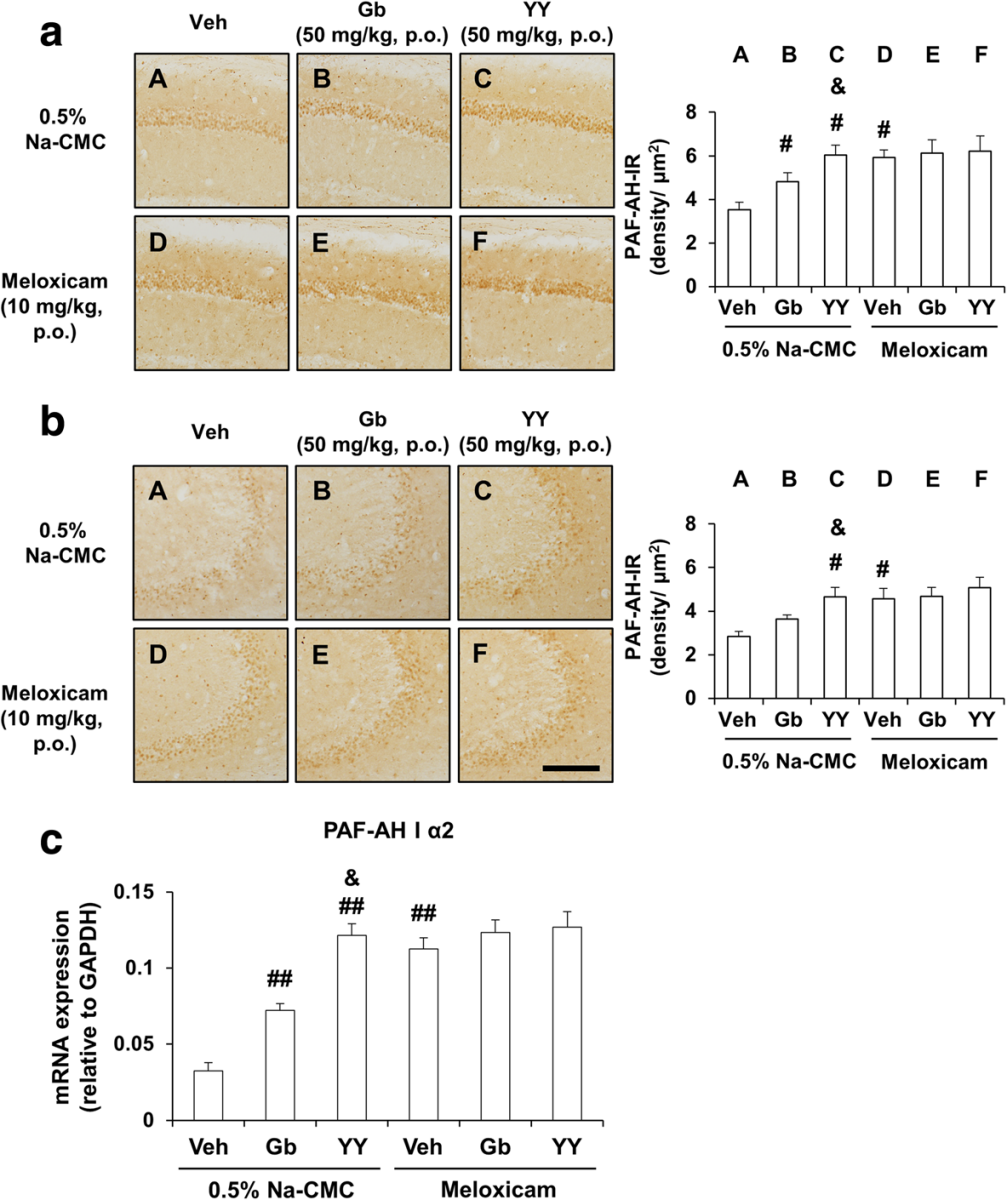
Activity of YY-1224 (YY) or Gb on PAF-AH level in the hippocampus of APP/PS1 Tg mice. **a**, **b** Effects of meloxicam on the pharmacological activity of YY or Gb in response to PAF-AH-immunoreactivity in the CA1 (**a**) and CA3 (**b**) regions of the hippocampus. **c** Effects of meloxicam on the pharmacological activity of YY or Gb in response to PAF-AH I α2 mRNA expression in the hippocampus. *Veh* vehicle for YY or Gb (10% tween-80 in sterile saline). Each value is the mean ± S.E.M of five animals. ^#^ *P* < 0.05, ^##^ *P* < 0.01 vs. vehicle + 0.5% Na-CMC; &*P* < 0.05 vs. Gb + 0.5% Na-CMC (two-way ANOVA was followed by Fisher’s LSD pairwise comparisons). *Scale bar* = 200 μm

Because YY-1224 exerted its pharmacological effect through the enhancement of PAF-AH I and PPARγ expression in Aβ (1-42)-treated mice, we examined the effect of YY-1224 on the mRNA and protein expression of PAF-AH I and PPARγ in the hippocampus of APP/PS1 Tg mice. In line with the results from Aβ-treated mice, ANOVA and post hoc pairwise comparisons indicated that PAF-AH I-immunoreactivity was significantly increased by YY-1224 (Fig. 10a, b, *P* < 0.05 for CA1 and CA3), Gb (Fig. 10a, *P* < 0.05 for CA1), and pharmacological inhibition (i.e., meloxicam) of COX-2 (Fig. 10a, b, *P* < 0.05 for CA1 and CA3) in APP/PS1 Tg mice. Since the α2 subunit of PAF-AH I was positively regulated by YY-1224 in Aβ-treated mice, we examined the mRNA expression of this subunit in APP/PS1 Tg mice. ANOVA and post hoc pairwise comparisons revealed that the mRNA expression of the PAF-AH I α2 subunit was significantly enhanced by YY-1224 (Fig. 10c, *P* < 0.01), Gb (Fig. 10c, *P* < 0.01), and COX-2 inhibition (i.e., meloxicam; Fig. 10c, *P* < 0.01). YY-1224 enhanced PAF-AH I-immunoreactivity and PAF-AH I α2 mRNA expression more than Gb (Fig. 10a–c, ANOVA and post hoc pairwise comparisons showing the difference between Gb and YY-1224, *P* < 0.05). The results from RT-rt-PCR of PAF-AH I α2 subunit mRNA expression were comparable to those from RT-PCR (Additional file 1: Figure S11a).

**Fig. 11:**
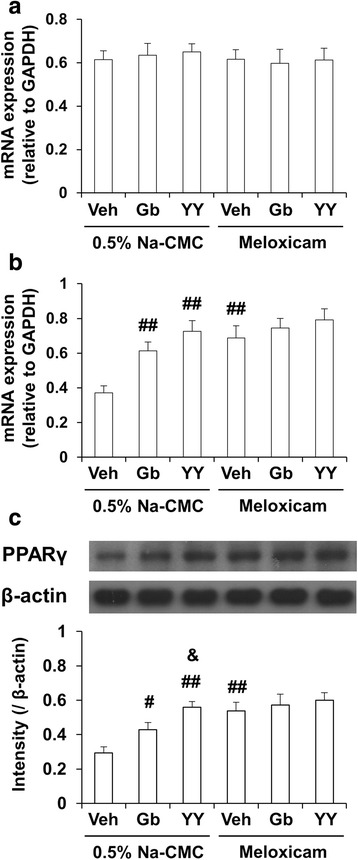
Activity of YY-1224 (YY) or Gb on PPAR expressions in the hippocampus of APP/PS1 Tg mice. **a** Effects of meloxicam on the pharmacological activity of YY or Gb in response to PPARα mRNA expression. **b**, **c** Effects of meloxicam on the pharmacological activity of YY or Gb in response to PPARγ mRNA (**b**) and protein (**c**) expression. *Veh* vehicle for YY or Gb (10% tween-80 in sterile saline). Each value is the mean ± S.E.M of five animals. ^#^ *P* < 0.05, ^##^ *P* < 0.01 vs. vehicle + 0.5% Na-CMC; &*P* < 0.05 vs. Gb + 0.5% Na-CMC (two-way ANOVA was followed by Fisher’s LSD pairwise comparisons)

As shown in Fig. 11, ANOVA and post hoc pairwise comparisons showed that the mRNA and protein expressions of PPARγ were significantly increased by treatment with YY-1224 (Fig. 11b, c, *P* < 0.01), Gb (Fig. 11b, c, *P* < 0.01 for mRNA, *P* < 0.05 for protein), or meloxicam (Fig. 11b, c, *P* < 0.01) in APP/PS1 Tg mice, and these results were consistent with the results from Aβ-treated mice. The pharmacological action of YY-1224 was more effective than that of Gb in PPARγ protein expression (Fig. 11c, ANOVA and post hoc pairwise comparisons showing the difference between Gb and YY-1224, *P* < 0.05). In addition, meloxicam did not affect the expression of PAF-AH or PPARγ in YY-1224- or Gb-treated APP/PS1 Tg mice (Figs. 10 and 11). The results from RT-rt-PCR of PPARα or PPARγ mRNA expression were comparable to those from RT-PCR (Additional file 1: Figure S11b and c).

### Effects of meloxicam on the pharmacological activity of YY-1224 in response to reactive microgliosis in APP/PS1 Tg mice

**Fig. 12:**
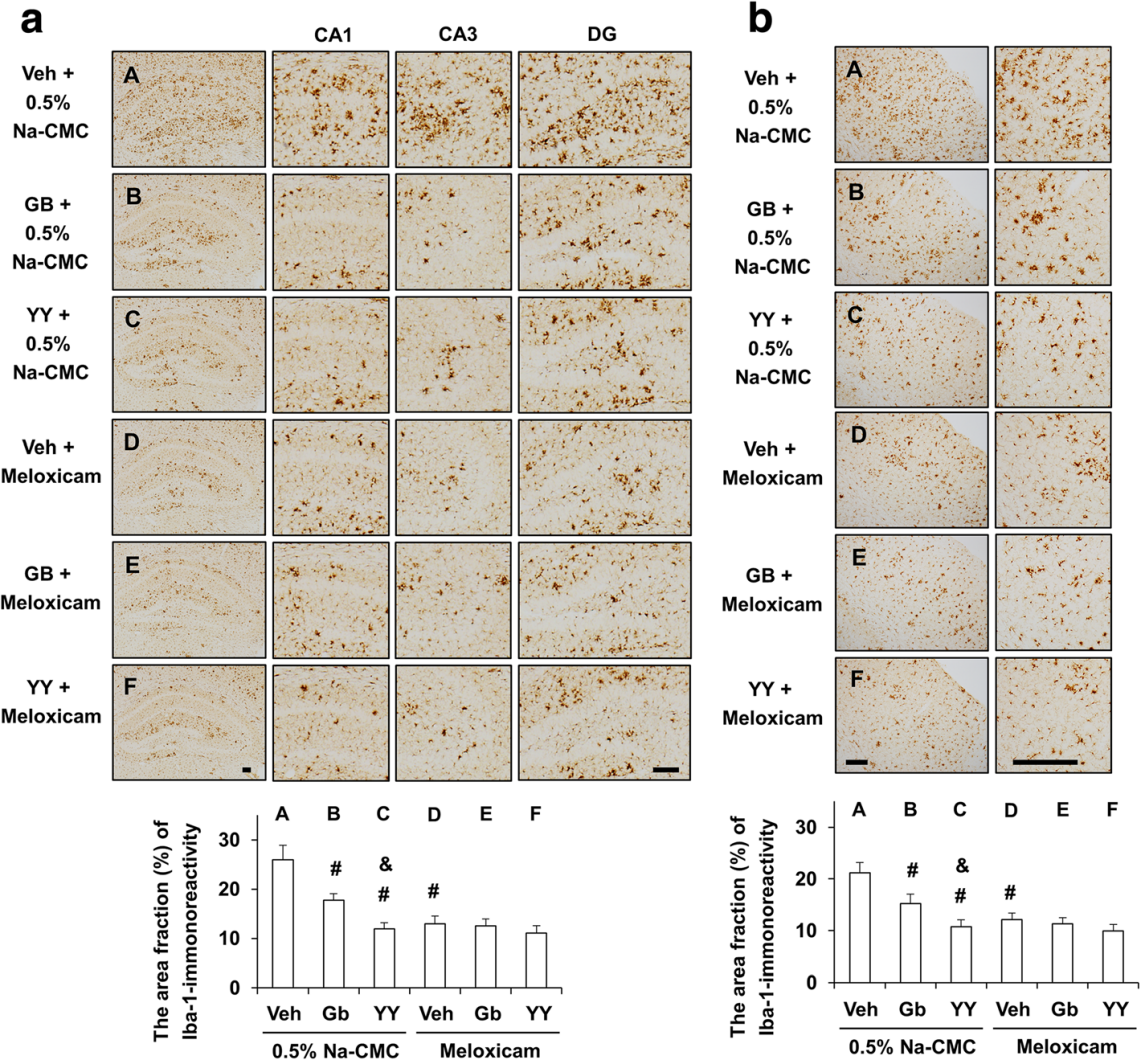
Activity of YY-1224 (YY) or Gb on Iba-1-immunoreactivity in the APP/PS1 Tg mice. **a**, **b** Effects of meloxicam on the pharmacological activity of YY or Gb in response to Iba-1-immunoreactivity in the hippocampus (**a**) and cortex (**b**). *Veh* vehicle for YY or Gb (10% tween-80 in sterile saline). Each value is the mean ± S.E.M of five animals. ^#^ *P* < 0.01 vs. vehicle + 0.5% Na-CMC; &*P* < 0.05 vs. Gb + 0.5% Na-CMC (two-way ANOVA was followed by Fisher’s LSD pairwise comparisons). *Scale bar* = 200 μm

To elucidate the role of microglia in the pharmacological effects of YY-1224, we examined the effect of Gb and YY-1224 on activated patterns of microglia (Fig. 12). Treatment with Gb and YY-1224 significantly attenuated Iba-1-labeled microglia in the hippocampus and cerebral cortex of 0.5% Na-CMC-treated APP/PS1 Tg mice (Fig. 12a, b, ANOVA and post hoc pairwise comparisons showing the inhibitory effect of YY-1224, Gb, and meloxicam, *P* < 0.01 for hippocampus and cortex). Meloxicam did not alter significantly the Iba-1 immunoreactivity mediated by YY-1224 or Gb in APP/PS1 Tg mice (Fig. 12).

**Fig. 13:**
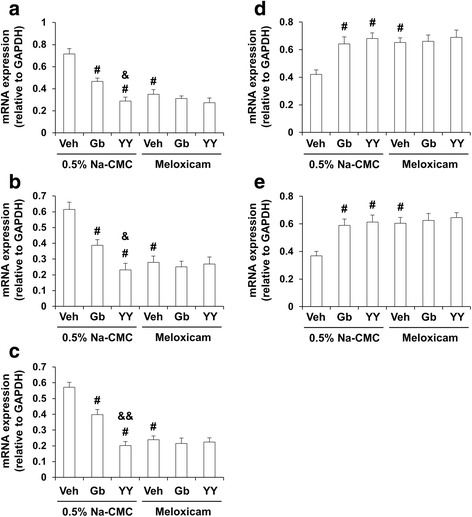
Activity of YY-1224 (YY) or Gb on mRNA expressions of microglial phenotype in the hippocampus. **a**–**c** Effect of meloxicam on the pharmacological activity of YY or Gb in response to CD16 (**a**), CD32 (**b**), and CD86 (**c**) mRNA expressions of M1 phenotype microglia/macrophages. **d**, **e** Effect of meloxicam on the pharmacological activity of YY or Gb in response to YM1 (**d**) and CD206 (**e**) mRNA expressions of M2 phenotype microglia/macrophages. *Veh* vehicle for YY or Gb (10% tween-80 in sterile saline). Each value is the mean ± S.E.M of five animals. ^*#*^ *P* < 0.01 vs. vehicle + 0.5% Na-CMC; &*P* < 0.05, &&*P* < 0.01 vs. Gb + 0.5% Na-CMC (two-way ANOVA was followed by Fisher’s LSD pairwise comparisons)

Different roles of microglia in tissue repair or damage may be due to distinct microglial subsets, i.e., “classically activated” pro-inflammatory (M1) or “alternatively activated” anti-inflammatory (M2) cells. Treatment with Gb or YY-1224 significantly decreased mRNA levels of M1 markers (CD16, CD32, and CD86) in the hippocampus of 0.5% Na-CMC-treated APP/PS1 Tg mice (Fig. 13a–c, ANOVA and post hoc pairwise comparisons showing the inhibitory effect of YY-1224 and Gb, *P* < 0.01). In contrast, Gb and YY-1224 significantly enhanced the mRNA levels of YM1 and CD206 of the M2 phenotype in the hippocampus of 0.5% Na-CMC-treated APP/PS1 Tg mice (Fig. 13d, e, ANOVA and post hoc pairwise comparisons showing the inhibitory effect of YY-1224 and Gb, *P* < 0.01). In meloxicam-treated groups, mRNA levels of CD16, CD32, and CD86 were significantly decreased in the hippocampus, while YM1 and CD206 mRNA levels were significantly increased (Fig. 13, ANOVA and post hoc pairwise comparisons showing the inhibitory effect of meloxicam, *P* < 0.01). In addition, meloxicam did not significantly alter the mRNA expression of CD16, CD32, CD86, YM1, and CD206 mediated by YY-1224 or Gb in APP/PS1 Tg mice (Fig. 13). The results from RT-rt-PCR of the mRNA expression of microglial phenotypic markers were comparable to those from RT-PCR (Additional file 1: Figure S12).

## Discussion

Increasing evidence has shown that Aβ-triggered microglial inflammatory activation damages neurons in AD pathogenesis [5, 57]. In this study, YY-1224 improved cognitive function and inhibited neuroinflammatory activation in COX-2 (+/+) mice after Aβ (1-42) treatment. YY-1224 inhibited the production of pro-inflammatory factors and oxidative stress through PAFR antagonism and activation of the PPARγ-COX-2 signaling pathway. YY-1224 appeared to be more effective than Gb against cognitive dysfunction and Aβ pathology in APP/PS1 Tg mice as well as against Aβ-induced memory impairment via microglia polarization. Previous studies have suggested that the antioxidant activity of Gb contributes to a memory-enhancing effect in AD patients or aged rats [58, 59]. Like NSAIDs, the constituents of Gb attenuated the production of inflammatory mediators such as COX-2, iNOS, and TNF-α in vitro [60]. We hypothesize that YY-1224 facilitates memory-enhancing and anti-inflammatory effects by increasing the ratio of terpenoids such as bilobalide and ginkgolides [36]. Bilobalide has been reported to exert protective effects on neurons [37–40], to facilitate synaptic transmission and plasticity in hippocampal subfields [61, 62], and to enhance spatial learning and memory [63]. Ginkgolide B exerts neuroprotection by alleviating neurotoxicity [42] and neuronal apoptosis [41], in addition to acting as a PAF antagonist [64].

PAF is a highly active phospholipid mediator of inflammation [65]. The initial step of PAF formation is activation of phospholipase A2 (PLA2) in a calcium-dependent manner, yielding lyso-PAF. During this step, arachidonic acid is also released and can be converted to its respective cyclooxygenase and lipoxygenase products. The lyso-PAF is then acetylated in position 2 of the glycerol backbone by a coenzyme A (CoA)-dependent acetyltransferase. The majority of PAF’s effects are attributed to interaction with PAFR, which is expressed by multiple peripheral cell types; however, the effects of PAF are regionally restricted to microglia subpopulations in the CNS [66]. The inhibition of PAF activity is mediated by a deacetylation reaction catalyzed by PAF-AH [19]. PAF-AH I, originally identified in the brain, consists of three subunits (α1, α2, and β), in which the α subunits provide the catalytic activity. The complex has received attention in part because the subunit that modulates enzymatic activity (the β subunit) is the product of the LIS1 gene. LIS1 expression may be related to the regulation of PAF AH activity in the brain [67]. PAF-AH I has been implicated in neuronal development, neuronal functions, Alzheimer's disease, bipolar disorder, and tolerance to hypoxia [68]. Although the most structurally well-characterized component of PAF AH I is the α1 subunit [69], a highly specific role of the α2 subunit for PAF hydrolysis has been recognized [70]. In this study, the α2 subunit was the most sensitive to Aβ (1-42) exposure or double transgenic overexpression of APP and PS1. Therefore, the α2 subunit may be a therapeutic target for YY-1224 or Gb in response to Aβ (1-42) exposure or double transgenic overexpression of APP and PS1via positive modulation of COX-2.

Among two major groups of constituents of YY-1224, flavonoid (24%) and terpenoid (12%) ginkgolides are known as potent antagonists of PAF [33]. YY-1224 treatment significantly attenuated decreases in the mRNA expression of PAF-AH I α2 and PAFR in response to Aβ (1-42) in COX-2 (+/+) mice. This suggests that the induction of PAF-AH I α2 and the PAF antagonistic activity of YY-1224 may contribute to its protective effects against Aβ (1-42) toxicity and possibly the double transgenic overexpression of APP and PS1.

Our current findings are consistent with previous reports that activation of PPARγ regulates the expression of PAF-AH [71]. We also observed that repeated treatment with YY-1224 attenuates the Aβ (1-42)-induced decrease in PPARγ and PAF-AH expression in COX-2 (+/+) but not COX-2 (−/−) mice, suggesting that the PPARγ-PAF-AH pathway may mediate the protective effects of YY-1224 via positive modulation of COX-2. This notion is supported by previous findings that PAF induces the expression of COX-2 [72]. The connection between PAF and PGE2 production by COX-2 has also been demonstrated in astrocytes. Stimulation of these cells with the non-hydrolyzable analog methylcarbamyl-PAF increased the secretion of PGE2, which was reduced by inhibitors of COX-2 but not COX-1 [73]. In this manner, PAF, which is a short-lived molecule due to its rapid degradation by PAF-AH, is able to have a long lasting effect on neurons through induction of COX-2. We found that the deleterious effect of Aβ (1-42) on brain oxidative stress and inflammatory markers are found in COX-2 expressing- but not COX-2-null mice. In addition, the protective effect of YY-1224 is present only in COX-2 expressing- but not COX-2 null mice or APP/PS1 Tg mice treated with the selective COX-2 inhibitor, meloxicam. This suggests an important role of the PPAR-PAF-COX-2 pathway in the deleterious effect of Aβ (1-42) as well as beneficial effects of ginkgolides and YY-1224. Inhibition of this pathway could reduce the formation of pro-inflammatory products of COX-2 such as prostaglandins resulting in decreased levels of pro-inflammatory mediators. We also found that COX-2 inhibition might interfere with a possible feedforward loop to amplify the inflammatory process by reducing PAF signaling through induction of its degrading enzyme, PAF-AH I, and reduction in expression of the PAF receptor, PAFR. This potential feedforward loop could exist in parallel with a previously described feedback loop, where pro-inflammatory mediators formed by COX-2 induce the expression of PAF-AH, resulting in reduced PAF and resolution of inflammation [74]. In this context, it is interesting to note that unlike PAF-AH I, PAF-AH II is induced by Aβ (1-42), and such induction is abolished in COX-2 null mice, although this enzyme is highly expressed in the liver and kidney [75]. More studies are needed to determine how COX-2 metabolites such as prostaglandins differentially regulate PAF-AH isoforms, resulting in propagation or modulation of inflammation.

Besides its effects on neurons, Aβ can activate microglial cells. This could lead to neuroinflammation and progression of neurodegeneration [76]. Microglia/macrophages are capable of undergoing phenotypic polarization to the M1 phenotype to produce pro-inflammatory cytokines, or to the M2 phenotype to produce anti-inflammatory cytokines [77, 78]. A prevalence of M1 over M2 microglia/macrophages has been reported in neurodegenerative pathologies, such as AD, and in the elderly brain [79]. In the present study, Aβ (1-42) induced an increase in inflammatory cytokines including TNF-α and IL-1β in microglia from the COX-2 (+/+) mice. PPARγ agonists are able to prevent the detrimental polarization of microglia, or switch the polarization of reactive microglia from the pro-inflammatory to the anti-inflammatory phenotype [87, 80]. Therefore, our results suggest that inhibiting the M1 pro-inflammatory phenotype or activating microglial polarization toward an M2 anti-inflammatory phenotype might contribute to the memory-enhancing effect of YY-1224.

The results of this study are consistent with the notion that YY-1224 is a potential PPARγ agonist that could be useful in the treatment of AD. PPARγ agonists have potent anti-inflammatory activity [81, 82], and activation prevents brain damage through an anti-inflammatory effect on endothelial cells, astrocytes, and microglia [83]. Activation of nuclear factor kappa-light-chain-enhancer of activated B cells (NF-κB) and COX-2 increases expression of pro-inflammatory cytokines [84–87], whereas inhibition of NF-κB suppresses the expression of COX-2 induced by Aβ (25-35) in PC12 cells [87]. In contrast to NF-κB, activation of PPARγ inhibits the expression of pro-inflammatory cytokines such as TNF-α, IL-1β, and IL-6 in vitro [88, 89]. Treatment with a PPARγ agonist 15dPGJ2 inhibited NF-κB signaling and the DNA-binding activity of NF-κB in macrophages [90, 91]. Moreover, treatment with another PPARγ agonist, pioglitazone, attenuates the production of Aβ plaque burden and glial inflammation in the APPV717I transgenic mice [83] and cognitive impairments in AD patients [92].

## Conclusions

In conclusion, we have demonstrated that terpene trilactone-activating *G. biloba* YY-1224 ameliorates AD pathogenesis by inhibiting oxidative stress and neuroinflammation via positive modulation of microglial activation. These protective effects of YY-1224 were mediated by its PAF antagonistic potential and PPARγ activity through the COX-2 regulatory signaling pathway. Thus, YY-1224 may be a potential candidate for development of novel drugs for AD-like neurodegenerative disorders.

## Additional files

Additional file 1: Supplemental figures. **Figure S1.** Representative HPLC chromatograms of ginkgo flavone glycosides and terpene trilactones. **Figure S2.** Experimental design for evaluating the effects of YY-1224 on Aβ (1-42)-induced learning impairments in COX-2 (+/+) and COX-2 (−/−) mice. **Figure S3.** Effects of YY-1224 or Gb on changes in the protein expression of BDNF, GDNF, NGF, or IGF-1 after k252a or JB-1 treatment in the hippocampus of the COX-2 (+/+) mice. **Figure S4.** Effects of YY-1224 or Gb on changes in SOD-1 or GPx-1 protein expression after DDC or MS treatment in the hippocampi of the COX-2 (+/+) mice. **Figure S5.** Effect of YY-1224 or Gb on changes in COX-2 mRNA expression in the hippocampi of the COX-2 (+/+)-mice and on changes in COX-2 protein expression in PC12 cells or mixed cortical cells after treatment with Aβ (1-42). **Figure S6.** Effect of YY-1224 or Gb on Aβ (1-42)-induced cell death in PC12 cells or mixed cortical cells. **Figure S7.** Effects of YY-1224 or Gb on Aβ (1-42)-induced memory impairment in COX-2 (+/+) and COX-2 (−/−) mice. **Figure S8**. Effects of YY-1224 or Gb on Aβ (1-42)-induced changes in PAFR and PAF-AH mRNA levels in the hippocampus. **Figure S9.** Effects of YY-1224 or Gb on Aβ (1-42)-induced pro-inflammatory genes in the hippocampi of mice. **Figure S10.** Effects of YY-1224 or Gb on Aβ (1-42)-induced PPAR mRNA expressions in the hippocampi of mice. **Figure S11.** Effects of YY-1224 or Gb on the mRNA level of PAF-AH I α2 and PPAR in the hippocampus of APP/PS1 Tg mice. **Figure S12.** Effects of YY-1224 or Gb on mRNA expressions of microglial phenotype markers﻿ in the hippocampus. **Figure S13.** Schematic illustration of the image analysis to quantify the area of Aβ deposition or Iba-1-immunoreactivity. Detailed figure legends are included in the additional file 2. (PDF 5615 kb)

Additional file 2: Supplemental information. (DOCX 74 kb)

## Abbreviations

4-HNE: 4-Hydroxy-2-nonenal
AD: Alzheimer’s disease
ANOVA: Analysis of variance
APP/PS1 Tg: APPswe/PS1dE9 transgenic
Aβ: β-amyloid
CNS: Central nervous system
CoA: Coenzyme A
COX: Cyclooxygenase
Gb: *G. biloba* extract
IFN-γ: Interferon gamma
IL-1β: Interleukin-1 beta
IL-6: Interleukin-6
iNOS: Inducible NO synthase
lysoPAF: 1-*O*-alkyl-sn-glycero-3-phosphocholine
MDA: Malondialdehyde
Na-CMC: Sodium carboxymethyl cellulose
NF-κB: Nuclear factor kappa-light-chain-enhancer of activated B cells
NO: Nitric oxide
NSAIDs: Nonsteroidal anti-inflammatory drugs
PAF: Platelet-activating factor
PAF-AH: PAF acetylhydrolase
PGE2: Prostaglandin E2
PLA2: Phospholipase A2
PPAR: Peroxisome proliferator-activated receptor
ROS: Reactive oxygen species
RT-PCR: Reverse transcription and polymerase chain reaction
TNF-α: Tumor necrosis factor alpha
