# Supplementary material for: YY-1224, a terpene trilactone-strengthened Ginkgo biloba, attenuates neurodegenerative changes induced by β-amyloid (1-42) or double transgenic overexpression of APP and PS1 via inhibition of cyclooxygenase-2
Source: J Neuroinflammation. 2017 Apr 27;14:94. doi: 10.1186/s12974-017-0866-x (PMC5408406; doi:10.1186/s12974-017-0866-x)
Supplement: Supplementary file 1 — Supplemental figures. Figure S1. Representative HPLC chromatograms of ginkgo flavone glycosides and terpene trilactones. Figure S2. Experimental design for evaluating the effects of YY-1224 on Aβ (1-42)-induced learning impairments in COX-2 (+/+) and COX-2 (−/−) mice. Figure S3. Effects of YY-1224 or Gb on changes in the protein expression of BDNF, GDNF, NGF, or IGF-1 after k252a or JB-1 treatment in the hippocampus of the COX-2 (+/+) mice. Figure S4. Effects of YY-1224 or Gb on changes in SOD-1 or GPx-1 protein expression after DDC or MS treatment in the hippocampi of the COX-2 (+/+) mice. Figure S5. Effect of YY-1224 or Gb on changes in COX-2 mRNA expression in the hippocampi of the COX-2 (+/+)-mice and on changes in COX-2 protein expression in PC12 cells or mixed cortical cells after treatment with Aβ (1-42). Figure S6. Effect of YY-1224 or Gb on Aβ (1-42)-induced cell death in PC12 cells or mixed cortical cells. Figure S7. Effects of YY-1224 or Gb on Aβ (1-42)-induced memory impairment in COX-2 (+/+) and COX-2 (−/−) mice. Figure S8. Effects of YY-1224 or Gb on Aβ (1-42)-induced changes in PAFR and PAF-AH mRNA levels in the hippocampus. Figure S9. Effects of YY-1224 or Gb on Aβ (1-42)-induced pro-inflammatory genes in the hippocampi of mice. Figure S10. Effects of YY-1224 or Gb on Aβ (1-42)-induced PPAR mRNA expressions in the hippocampi of mice. Figure S11. Effects of YY-1224 or Gb on the mRNA level of PAF-AH I α2 and PPAR in the hippocampus of APP/PS1 Tg mice. Figure S12. Effects of YY-1224 or Gb on mRNA expressions of microglial phenotype markers﻿ in the hippocampus. Figure S13. Schematic illustration of the image analysis to quantify the area of Aβ deposition or Iba-1-immunoreactivity. Detailed figure legends are included in the additional file 2. (PDF 5615 kb) [file 12974_2017_866_MOESM1_ESM.pdf]

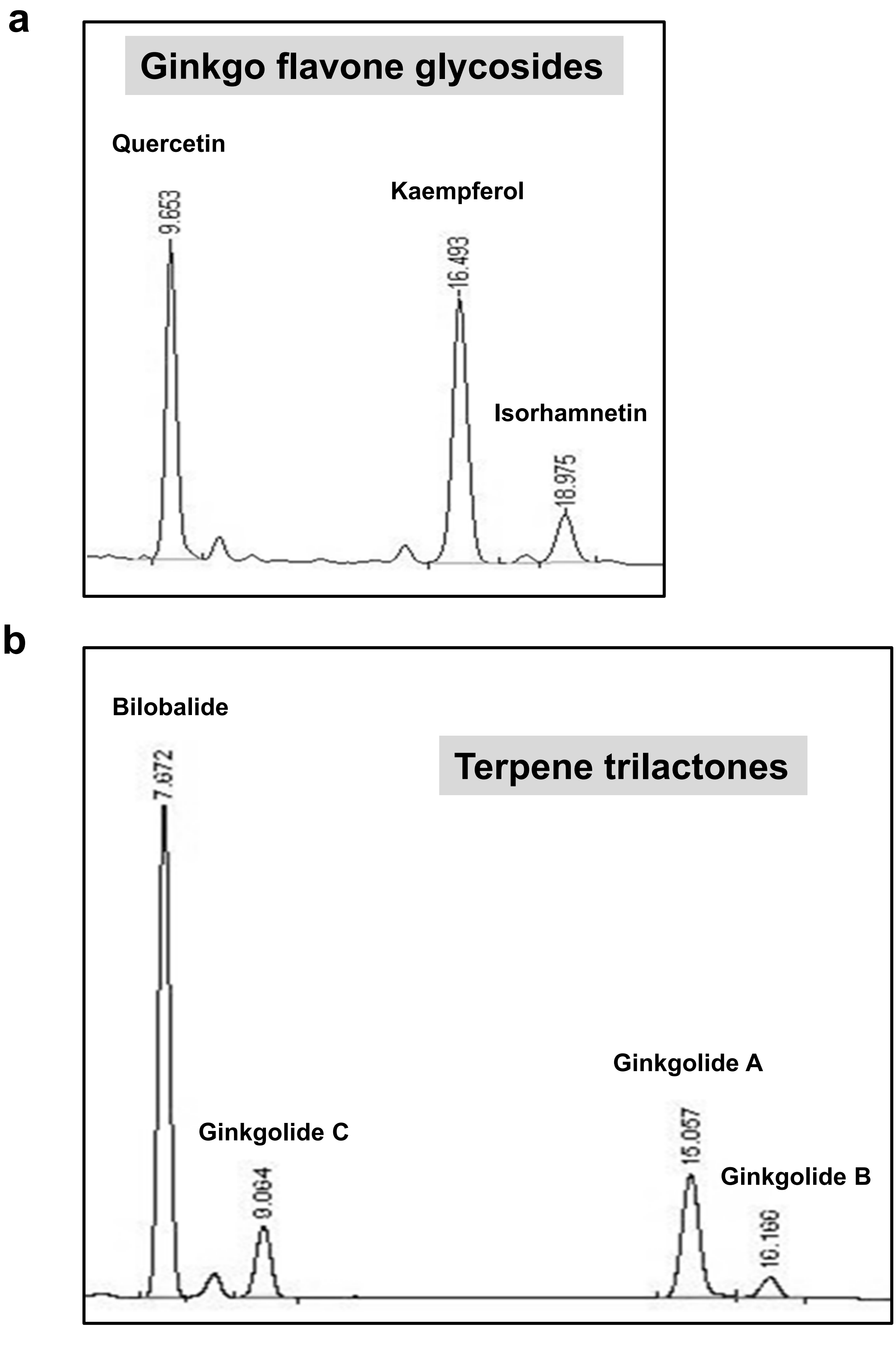

Supplemental Fig. 1

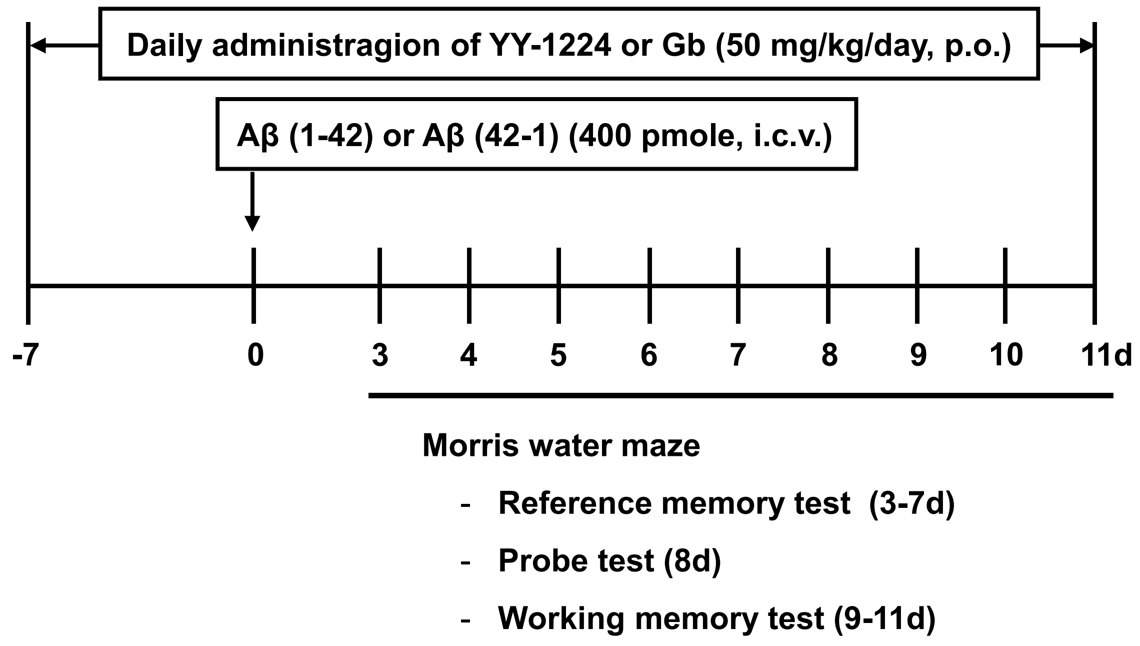

Supplemental Fig. 2

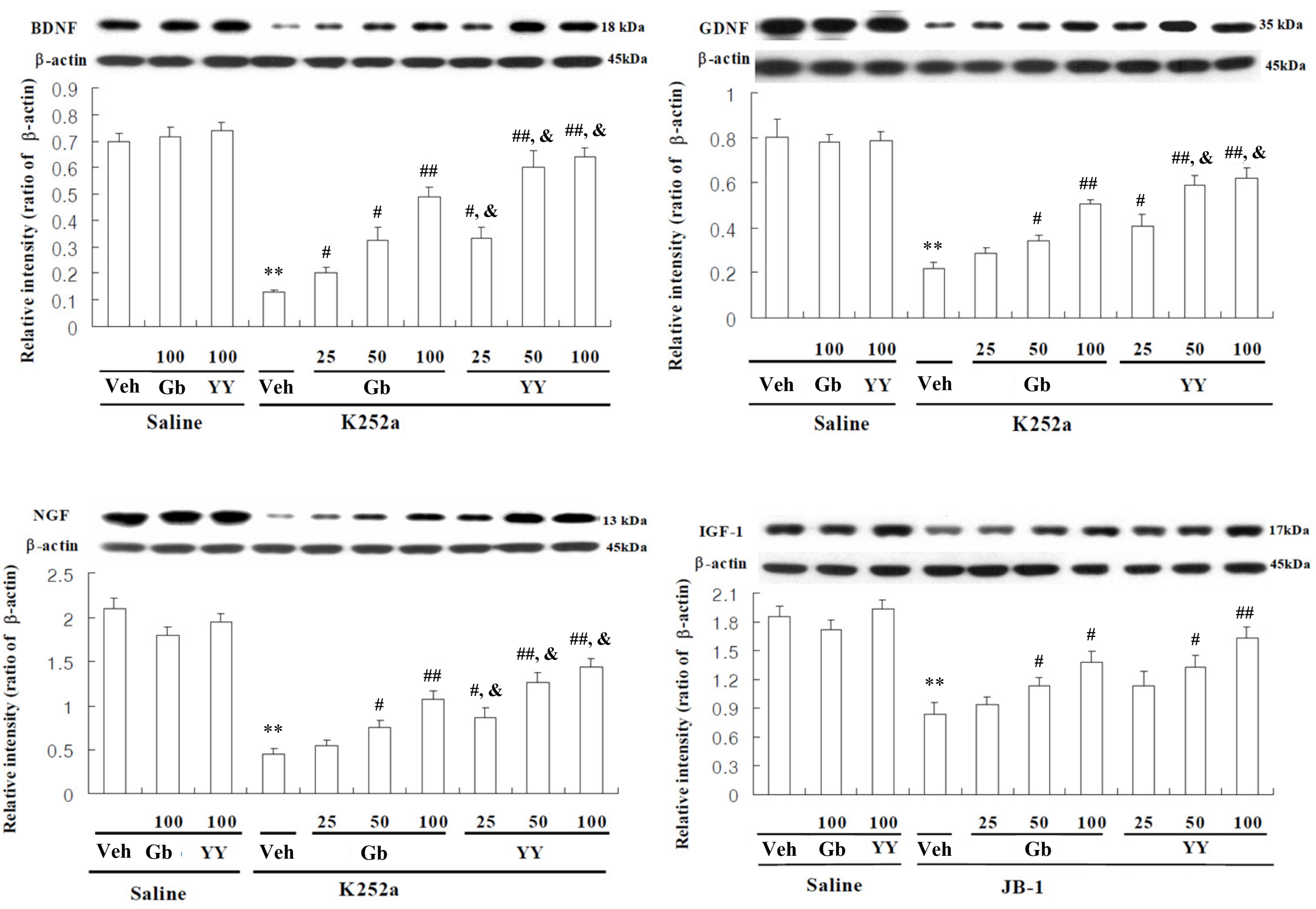

Supplemental Fig. 3

a

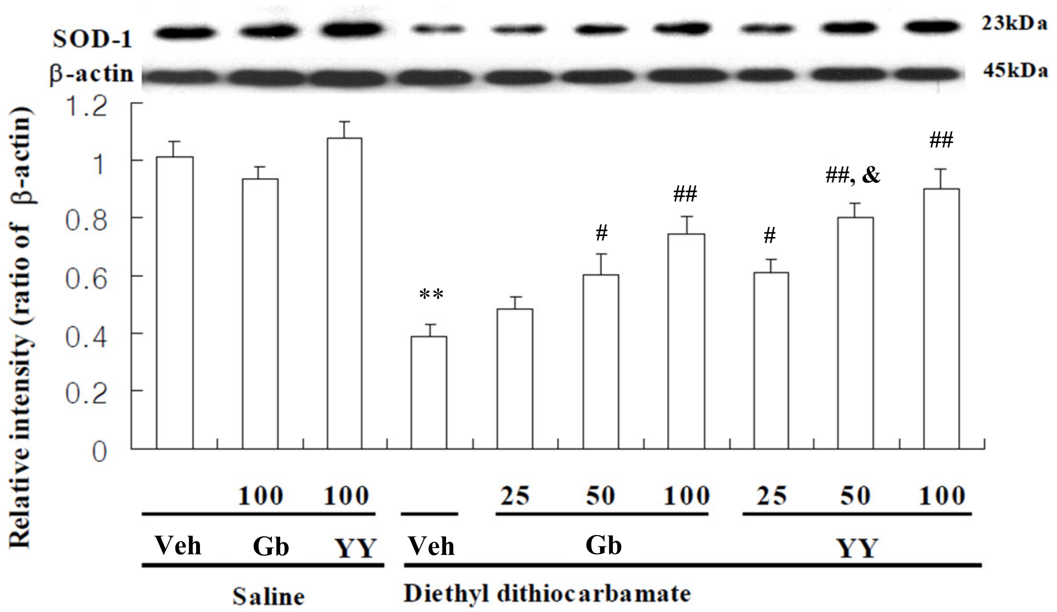

b

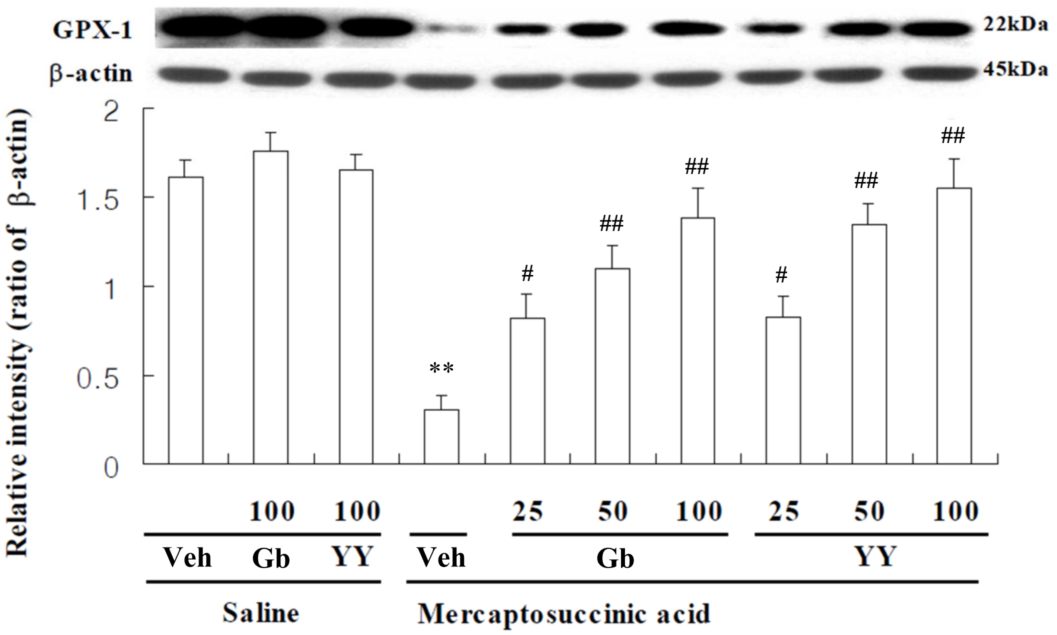

Supplemental Fig. 4

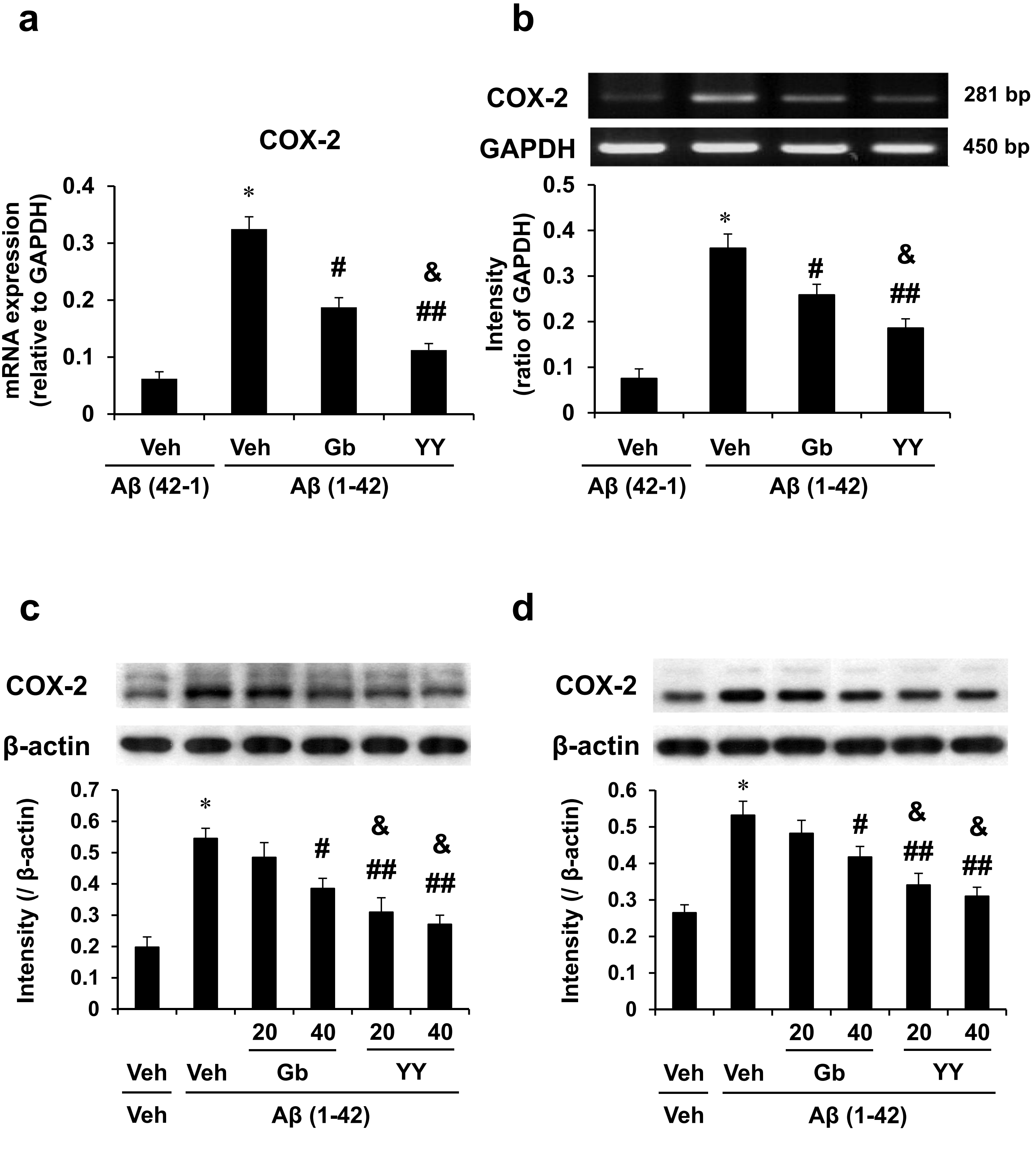

Supplemental Fig. 5

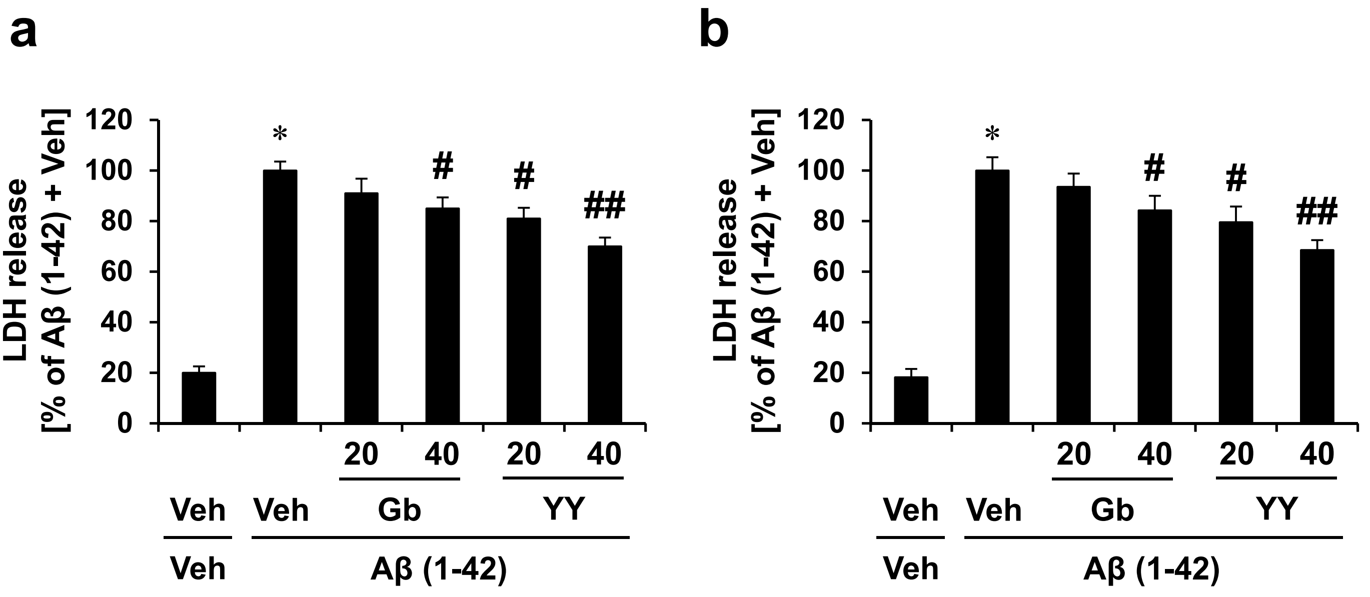

Supplemental Fig. 6

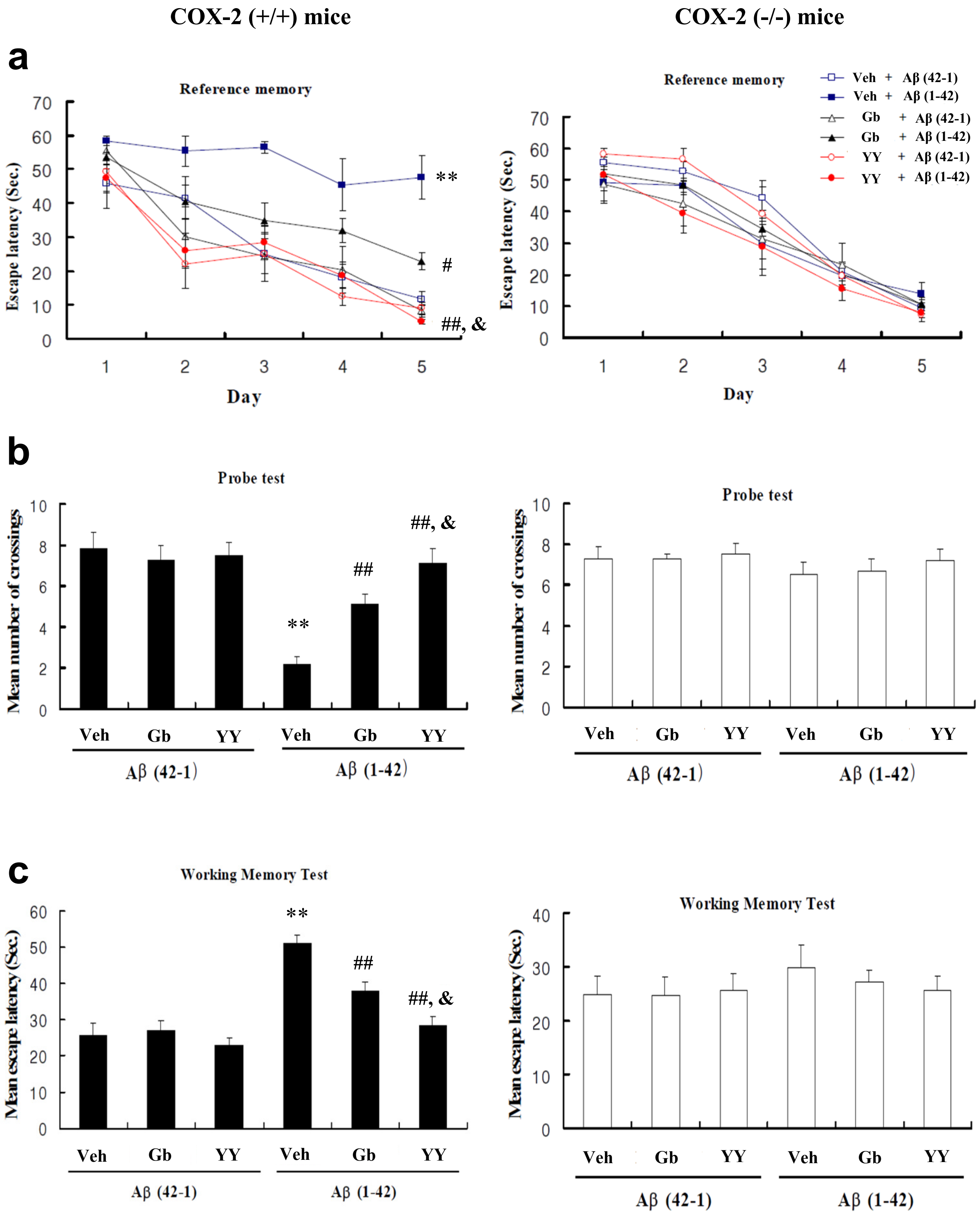

Supplemental Fig. 7

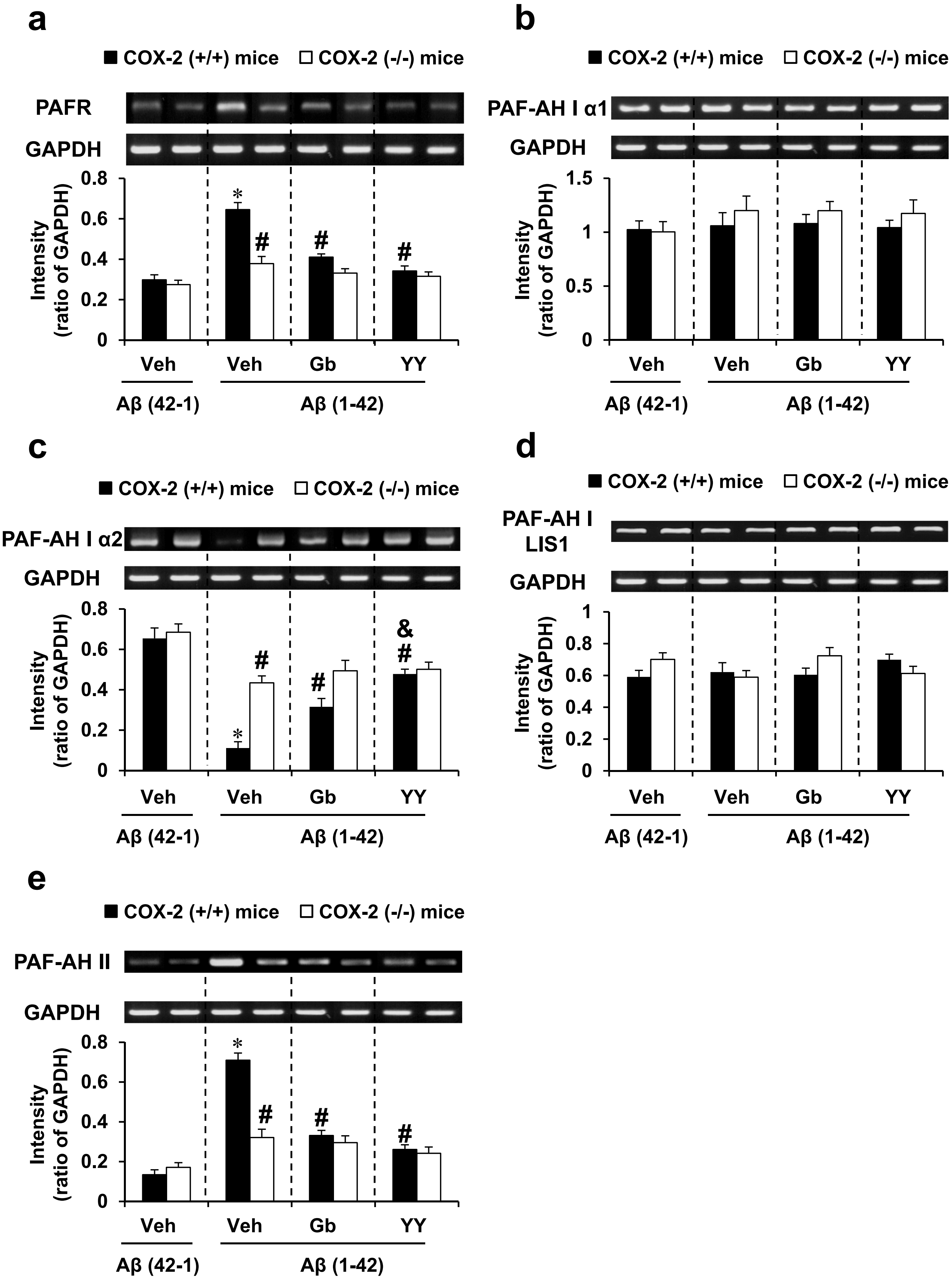

Supplemental Fig. 8

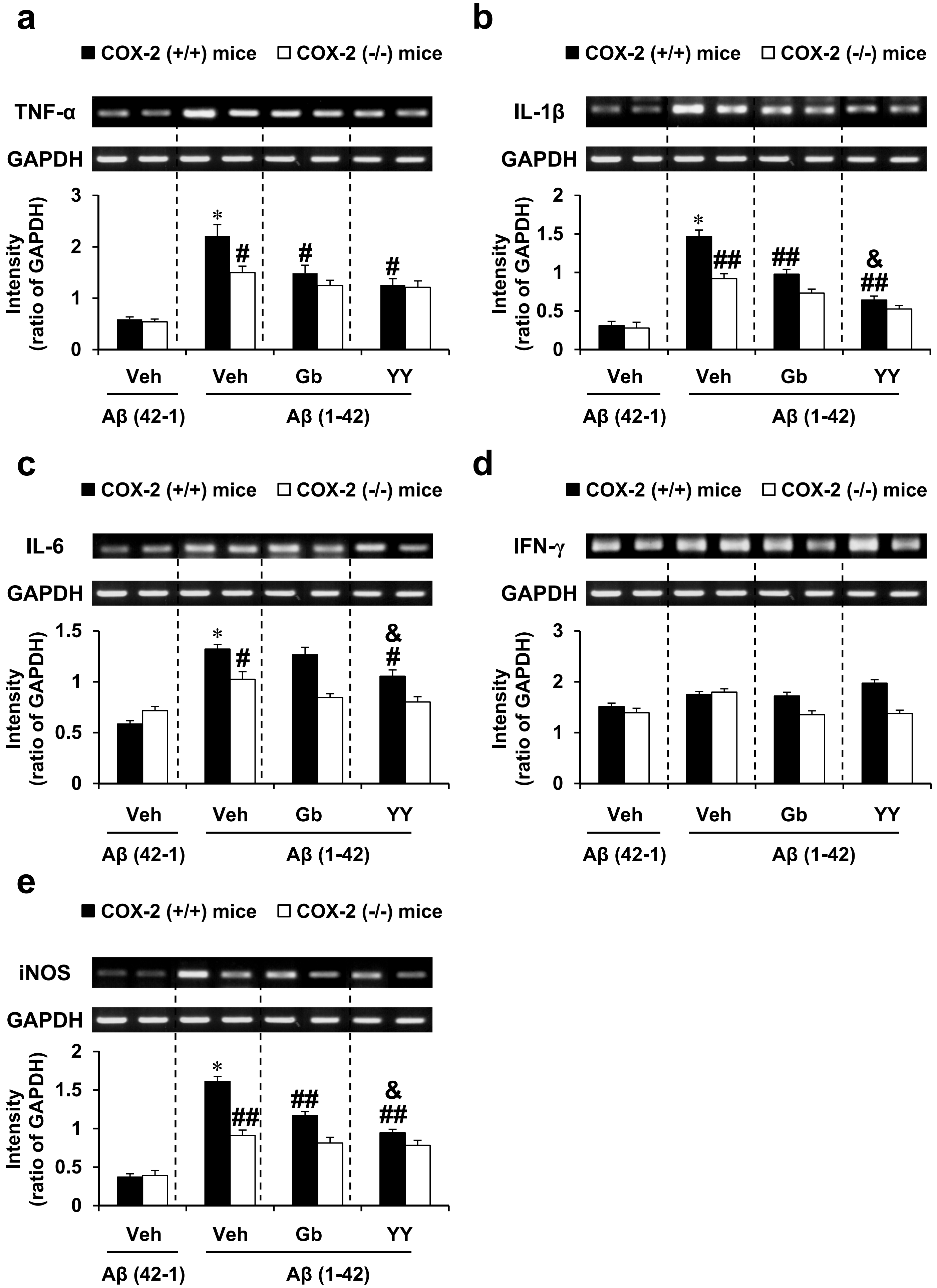

Supplemental Fig. 9

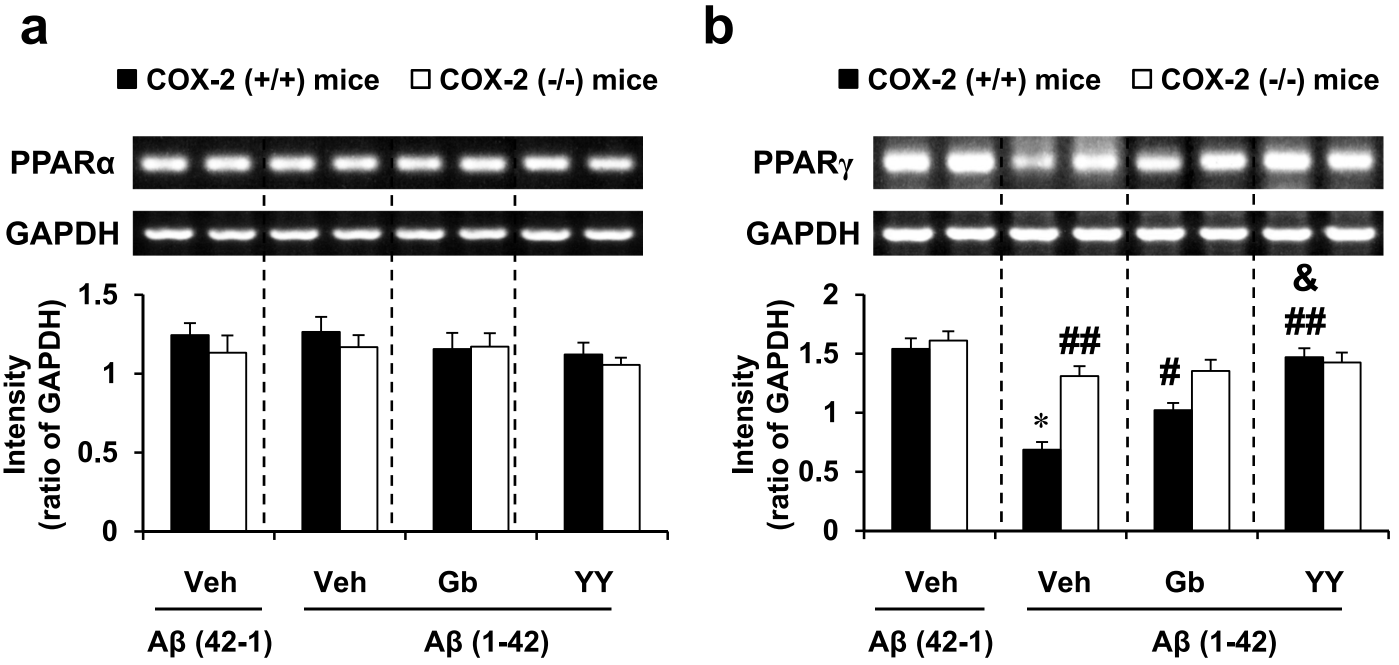

Supplemental Fig. 10

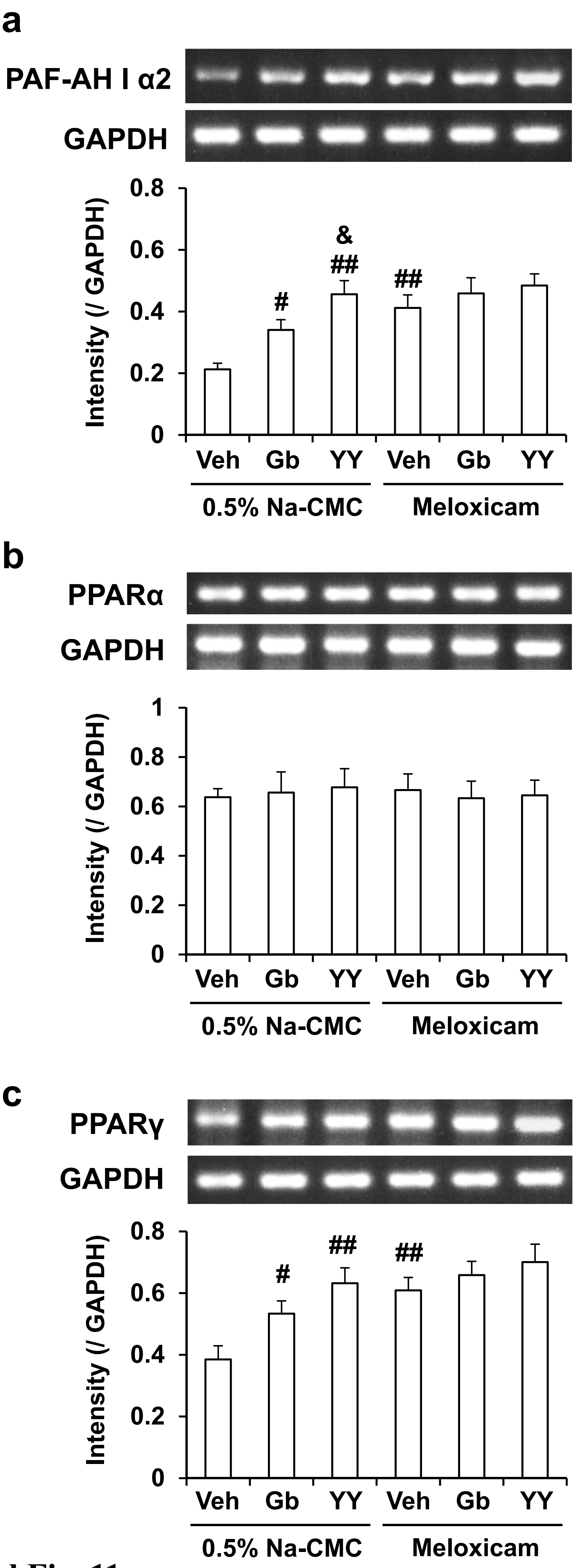

Supplemental Fig. 11

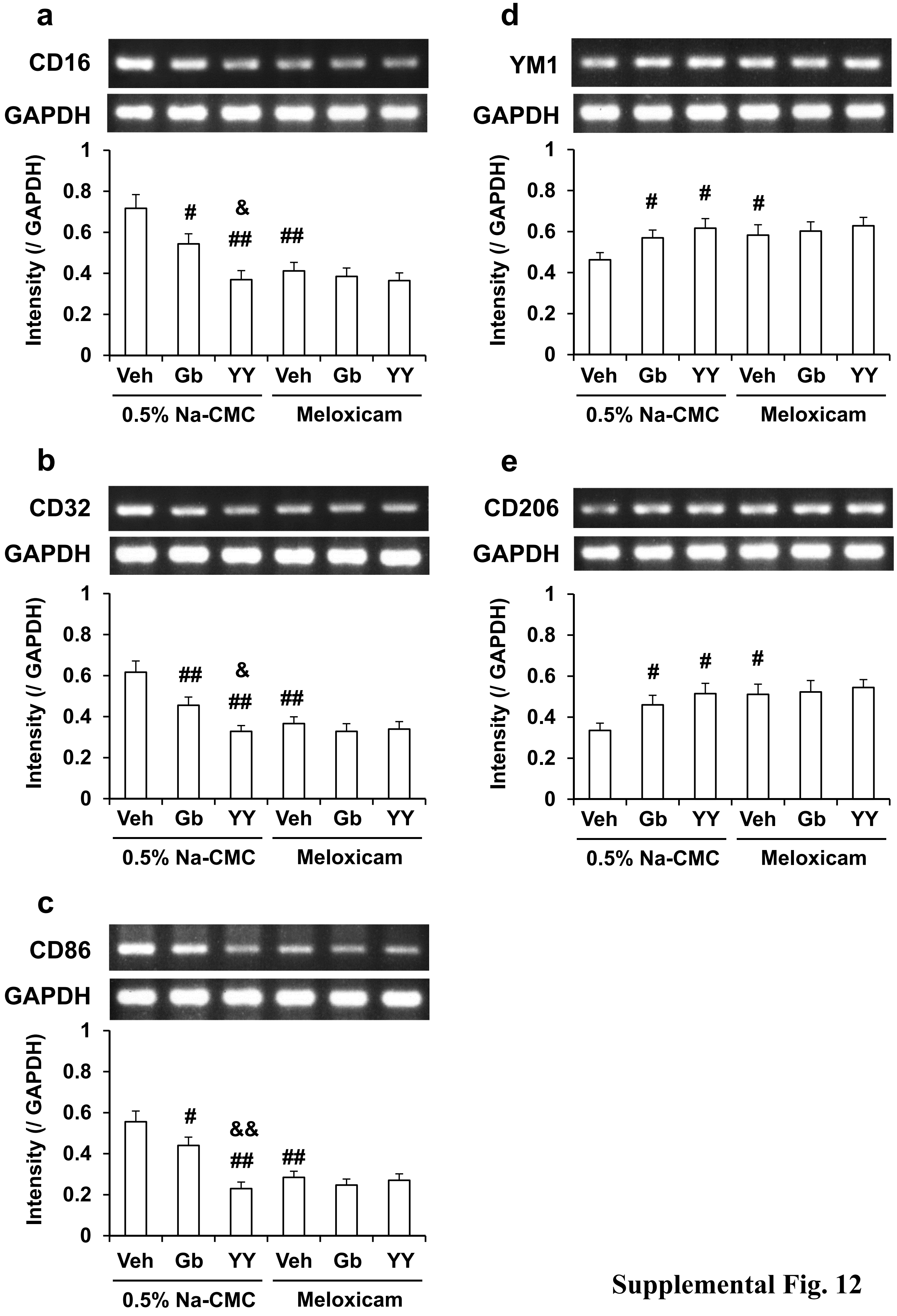

Supplemental Fig. 12

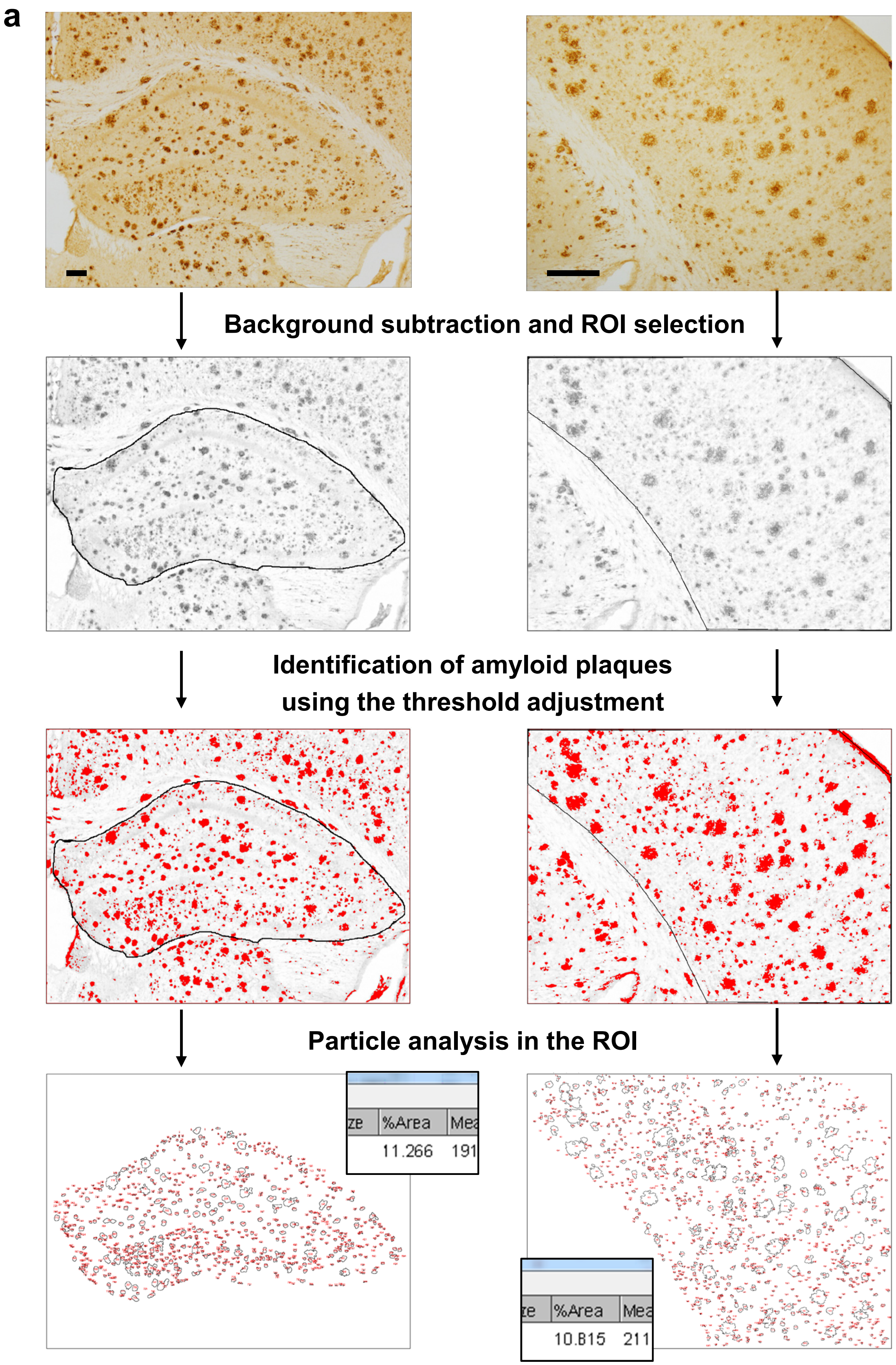

Supplemental Fig. 13

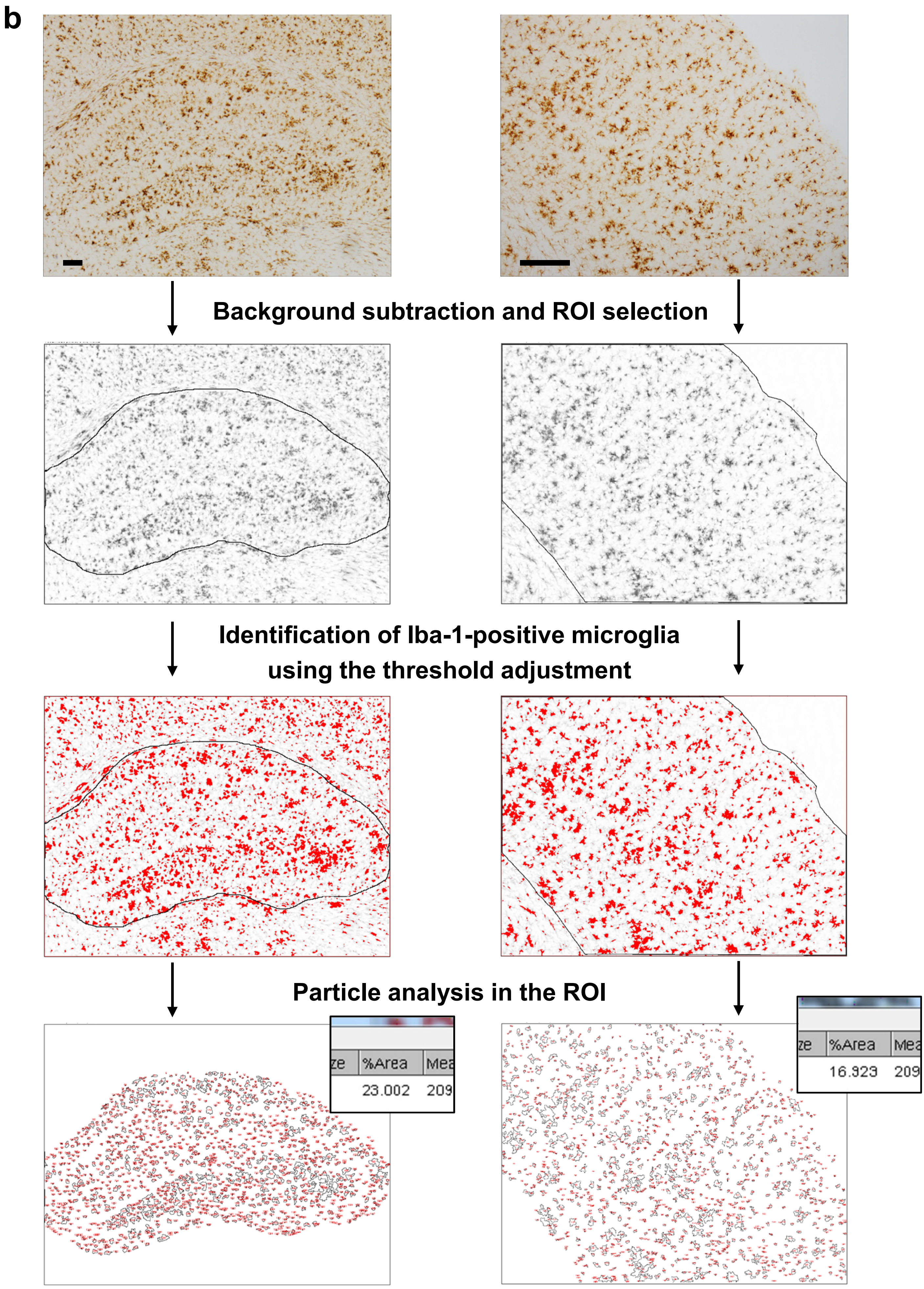

Supplemental Fig. 13 (continued)
