# Supplementary material for: YY-1224, a terpene trilactone-strengthened Ginkgo biloba, attenuates neurodegenerative changes induced by β-amyloid (1-42) or double transgenic overexpression of APP and PS1 via inhibition of cyclooxygenase-2
Source: J Neuroinflammation. 2017 Apr 27;14:94. doi: 10.1186/s12974-017-0866-x (PMC5408406; doi:10.1186/s12974-017-0866-x)
Supplement: Supplementary file 2 — Supplemental information. (DOCX 74 kb) [file 12974_2017_866_MOESM2_ESM.docx]

**Supplemental Information**

**Supplemental Materials and Methods**

**Cell culture and treatment**

Pheochromocytoma 12 (PC12) cells were cultured and maintained in Dulbecco’s modified Eagle’s medium (DMEM) supplemented with 10% (v/v) heat-inactivated fetal bovine serum (FBS) and 1% (v/v) penicillin/streptomycin (P/S) at 37 °C in 5% CO_2_ incubator. The cells (1 × 10^6^ cells/dish) were cultured in dishes pre-coated with poly-D-lysine (50 mg/ml in sterile water) overnight in DMEM supplemented with 1% FBS and 1% (P/S).

Mixed cortical cell cultures containing both glia and neurons were prepared from ICR mouse brains at 15-16 days of gestation. Briefly, dissociated neocortical cells (3.5 × 10^5^ cells/well) were plated onto primaria-coated 24-well plates (Falcon) containing a glial bed in plating medium consisting of Eagle's minimal essential medium (MEM; Earle's salts, supplied glutamine-free) supplemented with 20 mM glucose, 2 mM L-glutamine, 5% fetal bovine serum, and 5% horse serum.

First cells were pretreated with 20 or 40 µg/ml of Gb [diluted in dimethyl sulfoxide (DMSO); 0.2% as a final concentration] or YY-1224 (diluted in DMSO; 0.2% as a final concentration) for 2 h followed by Aβ (1-42) [25µM; diluted in phosphate buffered saline (PBS)] treatment for 24 h.

After the indicated treatments, supernatant was collected for lactate dehydrogenase (LDH) measurement and then cells were washed in PBS and lysed using lysis buffer containing 1 × phosphatase inhibitor cocktail I (Sigma-Aldrich) and 1 × protease inhibitor cocktail (Sigma-Aldrich). The lysate was centrifuged at 12,000 × g for 30 min and the supernatant fraction was used for western blot analysis.

**HPLC**

The HPLC instruments were equipped with an Agilent 1200 series bin pump, degasser, ALS autosampler, diode-array detector, and ELSD, and a Shiseido Nanospace series pump, degasser, autosampler, and UV–Vis detector [1]. For flavonoids, the column was a reverse-phase Zorbax XDB C18 (4.6 × 250 mm, 5 μm). The mobile phase was prepared by mixing water, methanol, and phosphoric acid in a ratio of 100:100:1. The analysis was performed at a flow rate of 1 mL/min. The UV detector was set at 270 nm and separation was performed isothermally at 35°C. The injection volume was 20 μL. A reverse-phase Capcellpak UG120 column (4.6 × 250 mm, 5 μm) was used for terpene lactones. The mobile phase consisted of solvent-A (H_2_O) and solvent-B (methanol) with the following gradient program: 30–40% B (0–23 min), 40–90% B (23–25 min), and 100% B isocratic (25–30 min). The analysis was performed at a flow rate of 1 mL/min. An ELSD detector was used and separation was performed isothermally at 35°C. The injection volume was 10 μL.

**Reverse transcription and conventional polymerase chain reaction (RT-PCR)**

RT-PCR was performed as described previously [2, 3]. Total RNA was isolated from the hippocampus using an RNeasy Mini Kit (Qiagen, Valencia, CA, USA) according to the manufacturer’s instructions. Reverse transcription reactions were carried out using the RNA to cDNA EcoDry Premix (Clontech, Palo Alto, CA, USA) with a 1-h incubation at 42°C. Primer sequences and PCR conditions are listed in Supplemental Table 1. PCR products were separated on 2% agarose gels containing ethidium bromide and visualized under ultraviolet light. Quantitative analysis of RNA was performed using PhotoCapt MW (version 10.01 for Windows; Vilber Lourmat).

**Drug treatment**

To examine the effect of YY-1224 on the level of neurotrophic factors and antioxidant enzymes, COX-2 (+/+) mice were treated with k252a (Tocris Bioscience, Ellisville, MO, U.S.A.), JB-1 (Bachem Bioscience, Inc., King of Prussia, PA, U.S.A.), diethyldithiocarbamate (DDC; Sigma-Aldrich, St. Louis, MO, U.S.A.), or mercaptosuccinic acid (MS; Sigma-Aldrich). A TrkB inhibitor k252a was dissolved in 5% (v/v) dimethyl sulfoxide (DMSO) in sterile saline immediately before use. JB-1, a selective antagonist of insulin growth factor-1 receptor (IGF-1R), DDC, a superoxide dismutase-1 (SOD-1) inhibitor, and MS, a glutathione peroxidase (GPX) inhibitor were dissolved in sterile saline. One hour after the final treatment with k252a (2 μg, i.c.v.), JB-1 (60 μg, i.c.v.), DDC (100 mg/kg, i.p., seven times at 12 h interval), or MS (30 mg/kg, i.p., three times at 12 h interval), COX-2 (+/+) mice received YY-1224 (25, 50, 100 mg/kg, p.o.) or Gb (25, 50, 100 mg/kg, p.o.), and were sacrificed 24 hours later.

**Western blot**

Western blot analysis was performed as described in the main text. The following antibodies were used in the supplemental experiment: anti-BDNF [1:500, Chemicon (EMD Millipore), Temecula, MA, U.S.A.], anti-GDNF (1:500, Santa Cruz Biotechnology, Inc., Santa Cruz, CA, U.S.A.), anti-NGF (1:200, Santa Cruz Biotechnology, Inc.), anti-IGF (1:5000, Abcam, Cambridge, MA, U.S.A.), anti-GPX-1 (1:1000, Ab Frontier, Seoul, Republic of Korea), anti-COX-2 (1:2000, Santa Cruz Biotechnology, Inc.), and anti-SOD-1 (1:1000; kindly gifted from Dr. Kanefusa Kato at Aichi Prefectural Colony, Kasugai, Japan) [4, 5].

**Morris water maze test**

The apparatus was a cylindrical water pool that was, 97 cm in diameter and 60 cm in height. During testing, the tank was filled with water (23 ± 2 °C) that was clouded with powdered milk. A transparent platform was set inside the tank and its top was submerged 2 cm below the water surface in the center of one quadrant of the maze. The tank was located in a large room with many extramaze cues that were constant throughout the study [6, 7]. The movements of the animal in the tank were recorded and analyzed with a video tracking system (EthoVision, Noldus, The Netherlands).

- *Hidden platform test*

For each training trial, the mouse was put into the pool at one of the five positions, the sequence of the positions being selected randomly. The platform was located a constant position throughout the test period in the middle of one quadrant, equidistant from the center and edge of the pool. In each training session, the latency to escape on to the hidden platform was recorded. If the mouse found the platform, it was allowed to remain there for 10 sec and was then returned to its home cage. If the mouse was unable to find the platform within 60 sec, the training was terminated and a maximum score of 60 sec was assigned. Training was conducted 4 trials a day for 5 consecutive days, and the results were averaged across 4 trials per day [6, 7].

- *Probe test*

One day after the final trial of hidden platform test, two trials of probe test were performed. The platform was removed from the pool and each mouse was allowed to swim for 60 sec in the maze. The time spent in the target quadrant where the platform had been located was recorded and averaged across 2 trials [6, 7].

- *Working memory (repeated acquisition) test*

The working memory test was started 1 day after the probe test, and we conducted five trials a day for three consecutive days. The working memory test was procedurally similar to reference memory test except that the platform location was changed daily. The first trial of the day was an informative sample trial in which the mouse was allowed to swim to the platform in its new location. Spatial working memory was regarded as the mean escape latency from the second to fifth trials [6, 7].

**Statistics**

Data were analyzed using IBM SPSS ver. 21.0 (IBM, Chicago, IL, U.S.A.). Analysis of variance (ANOVA) was employed to statistically analyze the effect of Aβ treatment, pretreatment (YY-1224 or Gb), or COX-2 inhibition (COX-2 gene knockout or COX-2 inhibitor). ANOVA for repeated measures was performed to analyze the statistical significance of the hidden platform test in the Morris water maze. Post-hoc Fisher’s least significant difference pairwise comparisons tests were then conducted. P-values < 0.05 were considered to be significant.

**Supplemental Results**

**Representative HPLC chromatograms of ginkgo flavone glycosides and terpene lactones**

As shown in Supplemental Fig. 1, ginkgo flavone glycosides were detected and quantified as quercetin (*t*_R_ = 9.653 min), kaempferol (*t*_R_ = 16.493 min) and isorhamnetin (*t*_R_ = 18.975 min). Total terpene lactones were detected and quantified as bilobalide (*t*_R_ = 7.672 min), ginkgolide A (*t*_R_ = 15.057 min), ginkgolide B (*t*_R_ = 16.166 min) and ginkgolide C (*t*_R_ = 9.064 min).

**Anti-inflammatory, neurotrophic, and antioxidative pharmacological action of YY-1224 or Gb in COX-2 (+/+) mice**

Treatment with k252a (a TrkB inhibitor, 2 μg, i.c.v.), JB-1 (a selective IGF-1R antagonist, 60 μg, i.c.v.), DDC (diethyldithiocarbamate, a SOD-1 inhibitor, 100 mg/kg, i.p.), or MS (mercaptosuccinate, GPx-1 inhibitor, 30 mg/kg, i.p.) significantly decreased the protein expression of BDNF, GDNF, NGF, IGF-1, SOD-1 and GPX-1 in the hippocampus of COX-2 (+/+) mice (Supplemental Figs. 3 and 4, ANOVA and *post-hoc* pairwise comparisons showing the effect of k252a, JB-1, DDC, or MS, P < 0.01). The changes were significantly attenuated by treatment with Gb (25, 50 and 100 mg/kg) and YY-1224 (25, 50 and 100 mg/kg) in a dose-dependent manner. YY-1224 was had a more pronounced effect than Gb. Furthermore, the effect of YY-1224 (50 mg/.kg) is comparable to that of YY-1224 (100 mg/kg) (Supplemental Figs. 3 and 4).

**Effects of YY-1224 or Gb on the changes induced by Aβ (1-42) in the hippocampal expression of COX-2 in mice**

COX-2 mRNA expression as determined by RT-rt-PCR and RT-PCR was increased in the hippocampus of mice 12 d after Aβ (1-42) administration [Supplemental Fig. 5a and b, ANOVA and *post-hoc* pairwise comparisons showing the effect of Aβ (1-42), P < 0.01]. Treatment with YY-1224 or Gb significantly attenuated these increases (Supplemental Fig. 5a and b, ANOVA and *post-hoc* pairwise comparisons showing the inhibitory effect of YY-1224 or Gb, P < 0.01 or P < 0.05].

**Effects of YY-1224 or Gb on Aβ (1-42)-induced COX-2 protein expression in PC12 cells or in mixed cortical cells**

Treatment with Aβ (1-42) for 24 h significantly increased COX-2 protein expression in PC12 cells [Supplemental Fig. 5c, ANOVA and *post-hoc* pairwise comparisons showing the effect of Aβ (1-42), P < 0.01]. ANOVA and *post-hoc* pairwise comparisons indicated that the Aβ (1-42)-induced increase in COX-2 protein expression was significantly attenuated by YY-1224 (Supplemental Fig. 5c, 20 or 40 µg/ml of YY-1224, P < 0.01) or Gb (Supplemental Fig. 5c, 40 µg/ml of Gb, P < 0.05), and that YY-1224 was more effective than Gb in attenuating COX-2 expression (Supplemental Fig. 5c, 20 or 40 µg/ml of YY-1224, P < 0.05). The results obtained from PC12 cells were comparable to those from mixed cortical cells (Supplemental Fig. 5d).

**Effect of GB or YY against Aβ (1-42)-induced neurotoxicity in PC12 cells or in mixed cortical cells**

Exposure of PC12 cells to Aβ (1-42) for 24 h caused a significant increase in LDH release [Supplemental Fig. 6a, ANOVA and *post-hoc* pairwise comparisons showing the effect of Aβ (1-42), P < 0.01]. ANOVA and *post-hoc* pairwise comparisons indicated that the Aβ (1-42)-induced LDH release was significantly attenuated by pretreatment with YY-1224 (Supplemental Fig. 6a, 20 or 40 µg/ml of YY-1224, P < 0.05 or P < 0.01) or Gb (Supplemental Fig. 6a, 40 µg/ml of Gb, P < 0.05) for 2 h in PC12 cells. The attenuation effects of YY-1224 appeared to be more pronounced than those of Gb, although this difference was not statistically significant. Similarly, as shown in Supplemental Fig. 6b, the attenuation of Aβ (1-42)-induced LDH release by Gb (20 or 40 µg/ml) or YY-1224 (20 or 40 µg/ml) in PC12 cells was comparable to those in mixed cortical cell cultures.

**Effects of YY-1224 or Gb on Aβ (1-42)-induced memory impairment in spatial learning as assessed by the water maze task in COX-2 (+/+)-and COX-2 (-/-)-mice**

The changes in escape latency onto a hidden platform during the reference memory test are shown in Supplemental Fig. 7a. ANOVA and *post-hoc* pairwise comparisons showed that Aβ (1-42) injection significantly increased escape latency in COX-2 (+/+) mice (Supplemental Fig. 7a, P < 0.01), and this increase in escape latency was significantly attenuated by YY-1224 (Supplemental Fig. 7a, P < 0.01) or Gb (Supplemental Fig. 7a, P < 0.05). Consistent with the results from the novel object recognition test and the Y-maze test, the memory enhancing effect of YY-1224 was significantly more pronounced than that of Gb (Fig. 7a, ANOVA and *post-hoc* pairwise comparisons showing the difference between Gb and YY-1224, P < 0.05). However, there was no significant change among groups in COX-2 (-/-) mice. The probe test confirmed the memory enhancing effect of YY-1224 and Gb in COX-2 (+/+) mice (Supplemental Fig. 7b).

As shown in Supplemental Fig. 6c, ANOVA and *post-hoc* pairwise comparisons showed that Aβ (1-42) treatment significantly increased the escape latency of the spatial working memory test in COX-2 (+/+) mice (Supplemental Fig. 7c, P < 0.01), and repeated treatment with YY-1224 or Gb significantly attenuated this increase in escape latency (Supplemental Fig. 7c, YY-1224 and Gb, P < 0.01). Consistently, attenuation mediated by YY-1224 was significantly more prominent than attenuation mediated by Gb (Fig. 7c, ANOVA and *post-hoc* pairwise comparisons showed the difference between Gb and YY-1224, P < 0.05). In COX-2 (-/-) mice, Aβ (1-42) did not significantly alter performance in the spatial working memory test.

**Effects of YY-1224 or Gb on Aβ (1-42)-induced changes in the mRNA expression of PAFR and PAF-AH in the hippocampi of COX-2 (+/+)- and COX-2 (-/-)-mice**

As shown in Supplemental Fig. 8, the results from conventional RT-PCR of the mRNA of PAFR and PAF-AH subtypes support the results from RT-rt-PCR (Figs. 3b and 5). ANOVA and *post-hoc* pairwise comparisons showed that Aβ (1-42) infusion significantly increased the mRNA levels of PAFR (Supplemental Fig. 8a, P < 0.01) and PAF-AH II (Supplemental Fig. 8e, P < 0.01), but significantly decreased PAF-AH I α2 mRNA expression (Supplemental Fig. 8c, P < 0.01) in the hippocampi of COX-2 (+/+) mice. These changes were significantly reversed by YY-1224, Gb, or COX-2 gene knockout (Supplemental Fig. 8a, c and e, ANOVA and *post-hoc* pairwise comparisons showing the inhibitory effect of YY-1224, Gb, or COX-2 gene knockout, P < 0.01). YY-1224 appeared to be more effective in enhancing PAF-AH 1α2 mRNA expression than was Gb in COX-2 (+/+) mice (Supplemental Fig. 8c, ANOVA and *post-hoc* pairwise comparisons showed the difference between Gb and YY-1224, P < 0.05).

**Effects of YY-1224 or Gb on changes in the expression of pro-inflammatory cytokines (TNF-α, IL-1β, IL-6, and IFN-γ) and iNOS induced by Aβ (1-42) in the hippocampi of COX-2 (+/+)- and COX-2 (-/-)-mice**

As shown in Supplemental Fig. 9, the results from conventional RT-PCR of the mRNA of TNF-α, IL-1β, IL-6, IFN-γ and iNOS were consistent with the results from RT-rt-PCR (Fig. 7). ANOVA and *post-hoc* pairwise comparisons revealed that the mRNA expression of TNF-α, IL-1β, IL-6 and iNOS, but not IFN-γ, was significantly increased by Aβ (1-42) in COX-2 (+/+) mice (Supplemental Fig. 9a-c and e, P < 0.01), and these changes were significantly attenuated by YY-1224 (Supplemental Fig. 9a-c and e, P < 0.05 for TNF-α and IL-6, P < 0.01 for IL-1β and iNOS), Gb (Supplemental Fig. 9a, b and e, P < 0.05 for TNF-α, P < 0.01 for IL-1β and iNOS), or COX-2 gene depletion (Supplemental Fig. 9a-c and e, P < 0.05 for TNF-α and IL-6, P < 0.01 for IL-1β and iNOS). The effects of YY-1224 were more pronounced than those of Gb on the increases in IL-1β, IL-6 and iNOS induced by Aβ (1-42) in COX-2 (+/+) mice (Supplemental Fig. 9b, c and e, ANOVA and *post-hoc* pairwise comparisons showed the difference between Gb and YY-1224, P < 0.05 for IL-1β, IL-6, and iNOS). YY-1224 and Gb did not show additional effects compared to COX-2 gene depletion in the presence of Aβ (1-42) (Supplemental Fig. 9)

**Effects of YY-1224 or Gb on changes in PPARγ mRNA expression induced by Aβ (1-42) in the hippocampi of COX-2 (+/+) and COX-2 (-/-) mice**

As shown in Supplemental Fig. 10, the results from conventional RT-PCR of the mRNA of PPARα and PPARγ were parallel to results from RT-rt-PCR (Fig. 8a and b). ANOVA and *post-hoc* pairwise comparisons showed that PPARγ mRNA expression was significantly decreased by Aβ (1-42) in COX-2 (+/+) mice (Supplemental Fig. 10b, P < 0.01), and this change was significantly attenuated by YY-1224 (Supplemental Fig. 10b, P < 0.01), Gb (Supplemental Fig. 10b, P < 0.05), or COX-2 gene depletion (Supplemental Fig. 10b, P < 0.01). YY-1224 more effectively enhanced PPARγ mRNA expression after Aβ (1-42) infusion in COX-2 (+/+) mice (Supplemental Fig. 10b, ANOVA and *post-hoc* pairwise comparisons showed the difference between Gb and YY-1224, P < 0.05). YY-1224 and Gb did not show additional effects compared to COX-2 gene depletion in the presence of Aβ (1-42) (Supplemental Fig. 10).

**Effects of meloxicam on pharmacological activity mediated by YY-1224 through PAF-AH I and PPARγ expression in the hippocampi of APP/PS1 Tg mice**

As shown in Supplemental Fig. 11, the results from conventional RT-PCR of the mRNA of PAF-AH I α2 subunit, PPARα and PPARγ in the hippocampi of APP/PS1 Tg mice were consistent with the results from RT-rt-PCR (Figs. 10c, 11a and 11b). ANOVA and *post-hoc* pairwise comparisons indicated that mRNA expression of the PAF-AH I α2 subunit and PPARγ was significantly increased by YY-1224 (Supplemental Fig. 11a and c, P < 0.01), Gb (Supplemental Fig. 11a and c, P < 0.05), and pharmacological inhibition (i.e., meloxicam) of COX-2 (Supplemental Fig. 11a and c, P < 0.01) in the hippocampus of APP/PS1 Tg mice. YY-1224 more highly enhanced PAF-AH I α2 subunit mRNA expression than did Gb (Supplemental Fig. 11a, ANOVA and *post-hoc* pairwise comparisons showed the difference between Gb and YY-1224, P < 0.05). In addition, meloxicam did not affect the mRNA expression of the PAF-AH I α2 subunit or PPARγ in YY-1224- or Gb-treated APP/PS1 Tg mice (Supplemental Fig. 11).

**Effects of meloxicam on the pharmacological activity of YY-1224 in response to microglial differentiation into an M1 phenotype in the hippocampi of APP/PS1 Tg mice**

As shown in Supplemental Fig. 12, the results from conventional RT-PCR of the mRNA of M1/M2 microglial phenotypic markers in the hippocampi of APP/PS1 Tg mice were parallel to the results from RT-rt-PCR (Figs. 13). ANOVA and *post-hoc* pairwise comparisons revealed that the mRNA levels of M1 phenotypic markers (CD16, CD32 and CD86) were significantly decreased, while the mRNA levels of M2 phenotypic markers (YM1 and CD206) were significantly increased by treatment with YY-1224 (Supplemental Fig. 12, P < 0.01 for CD16, CD32 and CD86, P < 0.05 for YM1 and CD206), Gb (Supplemental Fig. 12, P < 0.01 for CD32, P < 0.05 for CD16, CD86, YM1 and CD206), or meloxicam (Supplemental Fig. 12, P < 0.01 for CD16, CD32 and CD86, P < 0.05 for YM1 and CD206) in the hippocampus of 0.5% Na-CMC-treated APP/PS1 Tg mice. YY-1224 was significantly more effective in inhibiting the mRNA expression of M1 phenotypic markers than Gb (Supplemental Fig. 12a-c, ANOVA and *post-hoc* pairwise comparisons showing the difference between Gb and YY-1224, P < 0.05 for CD16 and CD32, P < 0.01 for CD86). In addition, meloxicam did not significantly alter the mRNA expression of CD16, CD32, CD86, YM1 or CD206 mediated by YY-1224 or Gb in APP/PS1 Tg mice (Supplemental Fig. 12).

**References**

1. Nam Y, Shin EJ, Shin SW, Lim YK, Jung JH, Lee JH, Ha JR, Chae JS, Ko SK, Jeong JH, Jang CG, Kim HC (2014) YY162 prevents ADHD-like behavioral side effects and cytotoxicity induced by Aroclor1254 via interactive signaling between antioxidant potential, BDNF/TrkB, DAT and NET. Food Chem Toxicol 65:280-292. doi: 10.1016/j.fct.2013.12.046
2. Shin EJ, Chung YH, Le HL, Jeong JH, Dang DK, Nam Y, Wie MB, Nah SY, Nabeshima Y, Nabeshima T, Kim HC. Melatonin attenuates memory impairment induced by klotho gene deficiency via interactive signaling between MT2 receptor, ERK, and Nrf2-related antioxidant potential. Int J Neuropsychopharmacol. 2014; doi: 10.1093/ijnp/pyu105.
3. Dang DK, Shin EJ, Nam Y, Ryoo S, Jeong JH, Jang CG, Nabeshima T, Hong JS, Kim HC. Apocynin prevents mitochondrial burdens, microglial activation, and pro-apoptosis induced by a toxic dose of methamphetamine in the striatum of mice via inhibition of p47phox activation by ERK. J Neuroinflammation. 2016;13:12.
4. Kurobe N, Suzuki F, Kato K, Sato T (1990) Sensitive immunoassay of rat cu/zn superoxide dismutase: Concentrations in the brain, liver, and kidney are not affected by aging. Biomed Res 11:187-194.
5. Kim HC, Bing G, Jhoo WK, Ko KH, Kim WK, Suh JH, Kim SJ, Kato K, Hong JS (2000) Changes of hippocampal cu/zn-superoxide dismutase after kainate treatment in the rat. Brain Res 853:215-226.
6. Jhoo JH, Kim HC, Nabeshima T, Yamada K, Shin EJ, Jhoo WK, Kim W, Kang KS, Jo SA, Woo JI (2004) Beta-amyloid (1-42)-induced learning and memory deficits in mice: Involvement of oxidative burdens in the hippocampus and cerebral cortex. Behav Brain Res 155:185-196.
7. Hwang SH, Shin EJ, Shin TJ, Lee BH, Choi SH, Kang J, Kim HJ, Kwon SH, Jang CG, Lee JH, Kim HC, Nah SY (2012) Gintonin, a ginseng-derived lysophosphatidic acid receptor ligand, attenuates alzheimer's disease-related neuropathies: Involvement of non-amyloidogenic processing. J Alzheimers Dis 31:207-223.

## Supplemental figure legends

**Supplemental Fig. 1.** Representative HPLC chromatogram of ginkgo flavone glycosides (a)

and terpene trilactones (b).

**Supplemental Fig. 2.** Experimental design for evaluating the effects of YY-1224 on Aβ (1-42)-induced learning impairments assessed by Morris water maze test in COX-2 (+/+) and COX-2 (−/−) mice; comparison with Gb.

**Supplemental Fig. 3.** Effects of YY-1224 (YY) or Gb on changes in the protein expression of brain derived neurotrophic factor (BDNF), glia derived neurotrophic factor (GDNF), nerve growth factor (NGF), or insulin growth factor-1 (IGF-1) after k252a or JB-1 treatment in the hippocampus of the COX-2 (+/+) mice. Standard extract of *Ginkgo biloba* (Gb, 50 mg/kg, p.o.) was used as a control drug. Veh = Vehicle for YY or Gb (10% tween-80 in sterile saline). Each value is the mean ± S.E.M of 6 animals. ^**^P < 0.01 vs. Veh + Saline; ^#^P < 0.05, ^##^P < 0.01 vs. Veh + K252a or JB-1; ^&^P < 0.05 vs. corresponding dose of Gb + K252a or JB-1 (two-way ANOVA was followed by Fisher’s LSD pairwise comparisons). For more details, please refer to “Supplemental Materials and Methods”

**Supplemental Fig. 4.** Effects of YY-1224 (YY) or Gb on changes in SOD-1 (a) or GPx-1 (b) protein expression after DDC or MS treatment in the hippocampi of the COX-2 (+/+) mice. Standard extract of *Ginkgo biloba* (Gb, 50 mg/kg, p.o.) was used as a control drug. Veh = Vehicle for YY or Gb (10% tween-80 in sterile saline). Each value is the mean ± S.E.M of 6 animals. ^**^P < 0.01 vs. Veh + Saline; ^#^P < 0.05, ^##^P < 0.01 vs. Veh + DDC or MS; ^&^P < 0.05 vs. corresponding dose of Gb + DDC or MS (two-way ANOVA was followed by Fisher’s LSD pairwise comparisons). For more details, please refer to “Supplemental Materials and Methods”

**Supplemental Fig. 5.** Effect of YY-1224 (YY) or Gb on changes in COX-2 mRNA expression in the hippocampi of the COX-2 (+/+)-mice (a and b) and on changes in COX-2 protein expression in PC12 cells (c) or mixed cortical cells (d) after treatment with Aβ (1-42). COX-2 mRNA expression was examined by RT-rt-PCR (a) and conventional RT-PCR (b). Standard extract of *Ginkgo biloba* (Gb, 50 mg/kg, p.o.) was used as a control drug. Veh = Vehicle for YY or Gb (10% tween-80 in sterile saline for *in vivo* experiments or 0.2% in sterile PBS for *in vitro* experiments). Each value is the mean ± S.E.M of 6 animals (a and b) or 3 experiments (c and d). ^*^P < 0.01 vs. Veh + Aβ (42-1); ^#^P < 0.05, ^##^P < 0.01 vs. Veh + Aβ (1-42); ^&^P < 0.05 vs. corresponding Gb + Aβ (1-42) (two-way ANOVA was followed by Fisher’s LSD pairwise comparisons).

**Supplemental Fig. 6.** Effect of YY-1224 (YY) or Gb on Aβ (1-42)-induced cell death, as evaluated by LDH release, in PC12 cells (a) or mixed cortical cells (b). Standard extract of *Ginkgo biloba* (Gb, 50 mg/kg, p.o.) was used as a control. Vehicle for YY or Gb (0.2% in sterile PBS). Each value is the mean ± S.E.M of 3 experiments. ^*^P < 0.01 vs. Veh + Aβ (42-1); ^#^P < 0.05, ^##^P < 0.01 vs. Veh + Aβ (1-42) (two-way ANOVA was followed by Fisher’s LSD pairwise comparisons).

**Supplemental Fig. 7.** Effects of YY-1224 (YY) or Gb on Aβ (1-42)-induced memory impairment [as shown by the hidden platform test (a), probe test (b) and working memory test (c)] in COX-2 (+/+) and COX-2 (−/−) mice. Standard extract of *Ginkgo biloba* (Gb, 50 mg/kg, p.o.) was used as a control drug. Veh = Vehicle for YY or Gb (10% tween-80 in sterile saline). Each value is the mean ± S.E.M of 6 animals. ^**^P < 0.01 vs. Veh + Aβ (42-1); ^#^P < 0.05, ^##^P < 0.01 vs. Veh + Aβ (1-42); ^&^P < 0.05 vs. Gb + Aβ (1-42) (A: two-way ANOVA for repeated measures; B: two-way ANOVA. Fisher’s LSD pairwise comparisons were followed).

**Supplemental Fig. 8.** Effects of YY-1224 (YY) or Gb on Aβ (1-42)-induced changes in PAFR and PAF-AH mRNA levels in the hippocampus. The mRNA level of each gene was examined by conventional RT-PCR. (a) Changes in PAFR mRNA expression. (b) Changes in PAF-AH I α1 mRNA expression. (c) Changes in PAF-AH I α2 mRNA expression. (d) Changes in PAF-AH I LIS1 mRNA expression. (e) Changes in PAF-AH II mRNA expression. Veh = Vehicle for YY or Gb (10% Tween-80 in sterile saline). Each value is the mean ± S.E.M of 6 animals. ^*^P < 0.01 vs. COX-2 (+/+) mice treated with Vehicle + Aβ (42-1); ^#^P < 0.01 vs. COX-2 (+/+) mice treated with Vehicle + Aβ (1-42); ^&^P < 0.05 vs. COX-2 (+/+) mice treated with Gb + Aβ (1-42) (three-way ANOVA was followed by Fisher’s LSD pairwise comparisons).

**Supplemental Fig. 9.** Effects of YY-1224 (YY) or Gb on Aβ (1-42)-induced proinflammatory genes in the hippocampi of mice. The mRNA level of each gene was examined by conventional RT-PCR. (a) Changes in TNF-α mRNA expression. (b) Changes in IL-1β mRNA expression. (c) Changes in IL-6 mRNA expression. (d) Changes in IFN-γ mRNA expression. (e) Changes in iNOS mRNA expression. Veh = Vehicle for YY or Gb (10% Tween-80 in sterile saline). Each value is the mean ± S.E.M of 6 animals. ^*^P < 0.01 vs. COX-2 (+/+) mice treated with Vehicle + Aβ (42-1); ^#^P < 0.05, ^##^P < 0.01 vs. COX-2 (+/+) mice treated with Vehicle + Aβ (1-42); ^&^P < 0.05 vs. COX-2 (+/+) mice treated with Gb + Aβ (1-42) (three-way ANOVA was followed by Fisher’s LSD pairwise comparisons).

**Supplemental Fig. 10.** Effects of YY-1224 (YY) or Gb on Aβ (1-42)-induced PPAR mRNA expressions in the hippocampi of mice. The mRNA level of each gene was examined by conventional RT-PCR. (a) Changes in PPARα mRNA expression. (b) Changes in PPARγ mRNA expression. Veh = Vehicle for YY or Gb (10% Tween-80 in sterile saline). Each value is the mean ± S.E.M of 6 animals. ^*^P < 0.01 vs. COX-2 (+/+) mice treated with Vehicle + Aβ (42-1); ^#^P < 0.05, ^##^P < 0.01 vs. COX-2 (+/+) mice treated with Vehicle + Aβ (1-42); ^&^P < 0.05 vs. COX-2 (+/+) mice treated with Gb + Aβ (1-42) (three-way ANOVA was followed by Fisher’s LSD pairwise comparisons).

**Supplemental Fig. 11.** Effects of YY-1224 (YY) or Gb on the mRNA level of PAF-AH I α2 and PPAR in the hippocampus of APP/PS1 Tg mice. The mRNA level of each gene was examined by conventional RT-PCR. (a) Changes in PAF-AH I α2 mRNA expression. (b) Changes in PPARα mRNA expression. (c) Changes in PPARγ mRNA expression. Veh = Vehicle for YY or Gb (10% Tween-80 in sterile saline). Each value is the mean ± S.E.M of 5 animals. ^#^P < 0.05, ^##^P < 0.01 vs. Vehicle + 0.5% Na-CMC; ^&^P < 0.05 vs. Gb + 0.5% Na-CMC (two-way ANOVA was followed by Fisher’s LSD pairwise comparisons).

**Supplemental Fig. 12.** Effects of YY-1224 (YY) or Gb on mRNA expressions of microglial phenotype in the hippocampus. The mRNA level of each gene was examined by conventional RT-PCR. (a-c) Effect of meloxicam on the pharmacological activity of YY or Gb in response to CD16 (a), CD32 (b), and CD86 (c) mRNA expression of M1 phenotype microglia/macrophages. (d-e) Effect of meloxicam on the pharmacological activity of YY or Gb in response to YM1 (d) and CD206 (e) mRNA expression of M2 phenotype microglia/macrophages. Veh = Vehicle for YY or Gb (10% Tween-80 in sterile saline). Each value is the mean ± S.E.M of 5 animals. ^#^P < 0.05, ^##^P < 0.01 vs. Vehicle + 0.5% Na-CMC; ^&^P < 0.05, ^&&^P < 0.01 vs. Gb + 0.5% Na-CMC (two-way ANOVA was followed by Fisher’s LSD pairwise comparisons).

**Supplemental Fig. 13.** Schematic illustration of the image analysis to quantify the area of Aβ deposition or Iba-1-immunoreactivity. (a) Quantification of the area fraction of Aβ deposition. (b) Quantification of the area fraction of Iba-1-immunoreactivity. ROI = region of interest. Scale bar = 200 μm.

**Supplemental Table 1.** Gene primer sequences and PCR conditions for RT-PCR analysis

| **Gene** | **Primer sequences (5’- 3’)** | **PCR condition**  **(denature/ annealing/ extension)** | **Cycles** | **Expected size (bp)** |
| --- | --- | --- | --- | --- |
| PAFR | F: CCTAGTGCCCAATAAGGATGGCT  R: TAGGAGTCTGGTTGGCTGGC | 94°C, 1 min/ 65°C, 30 sec/ 72°C, 2 min | 30 | 530 bp |
| PAF-AH I α1 | F: TCTGCATGCACTTAACTTTG  R: GGCTCAGGTGTAGGTAATCA | 94°C, 30 sec/ 55°C, 1 min/ 72°C, 2 min | 35 | 401 bp |
| PAF-AH I α2 | F: TGCAGCAGTACGAGATATGG  R: AACATGTCGTGGCAGGAGAT | 94°C, 30 sec/ 55°C, 1 min/ 72°C, 2 min | 40 | 418 bp |
| PAF-AH I LIS1 | F: AAGTATGCTGGTCTTTTGGA  R: AACCCTGAAAATCCCATAAT | 94°C, 30 sec/ 55°C, 1 min/ 72°C, 2 min | 33 | 409 bp |
| PAF-AH II | F: GACTGGATCTGATGACCTTG  R: TCCATTGATCGTAGTCTTCC | 94°C, 30 sec/ 55°C, 1 min/ 72°C, 2 min | 39 | 468 bp |
| TNF-α | F: AGCACAGAAAGCATGATCCG  R: ACTCCAGCTGCTCCTCCACT | 94°C, 30 sec/ 58°C, 30 sec/ 72°C, 1 min | 32 | 316 bp |
| IL-1β | F: GTTGACGGACCCCAAAAGAT  R: TCGTTGCTTGGTTCTCCT TG | 94°C, 30 sec/ 55°C, 1 min/ 72°C, 2 min | 35 | 440 bp |
| IL-6 | F: TGGAGTCACAGAAGGAGTGGCTAAG  R: TCTGACCACAGTGAGGAATGTCCAG | 94°C, 30 sec/ 59°C, 30 sec/ 72°C, 1 min | 35 | 155 bp |
| INF-γ | F: AGGAACTGGCAAAAGGATGGTG  R: CTGGACCTGTGGGTTGTTGA | 94°C, 30 sec/ 58°C, 30 sec/ 72°C, 30 sec | 33 | 219 bp |
| iNOS | F: GGCAAACCCAAGGTCTACGT  R: CTCACCATTATCTTTACTCAGTG | 94°C, 30 sec/ 55°C, 30 sec/ 72°C, 30 sec | 35 | 352 bp |
| PPAR-α | F: CCCTGAACATCGAGTGTCGAA  R: TTGCAGCTCCGATCACACTT | 94°C, 30 sec/ 55°C, 1 min/ 72°C, 2 min | 35 | 142 bp |
| PPAR- γ | F: TGTCGGTTTCAGAAGTGCCTT  R: GCTCGCAGATCAGCAGACTCT | 94°C, 30 sec/ 53°C, 30 sec/ 72°C, 30 sec | 35 | 146 bp |
| YM1 | F: ACCCCTGCCTGTGTACTCACCT  R: CACTGAACGGGGCAGGTCCAAA | 94°C, 1 min/ 60°C, 2 min/ 72°C, 1 min | 35 | 183 bp |
| CD206 | F: TCTTTGCCTTTCCCAGTCTCC  R: TGACACCCAGCGGAATTTC | 94°C, 1 min/ 60°C, 2 min/ 72°C, 1 min | 35 | 241 bp |
| CD16 | F: TTTGGACACCCAGATGTTTCAG  R: GTCTTCCTTGAGCACCTGGATC | 94°C, 1 min/ 60°C, 2 min/ 72°C, 1 min | 35 | 163 bp |
| CD32 | F: AATCCTGCCGTTCCTACTGATC  R: GTGTCACCGTGTCTTCCTTGAG | 94°C, 1 min/ 60°C, 2 min/ 72°C, 1 min | 35 | 187 bp |
| CD86 | F: TTGTGTGTGTTCTGGAAACGGAG  R: AACTTAGAGGCTGTGTTGCTGGG | 94°C, 1 min/ 60°C, 2 min/ 72°C, 1 min | 35 | 202 bp |
| COX-2 | F: ACACACTCTATCACTGGCACC  R: GTACGGCTTCAGGGAGAA | 94°C, 30 sec/ 55°C, 30 sec/ 72°C, 30 sec | 36 | 281 bp |
| GAPDH | F: ACCACAGTCCATGCCATCAC  R: TCCACCACCCTGTTGCTGTA | 94°C, 30 sec/ 55°C, 30 sec/ 72°C, 30 sec | 35 | 450 bp |
